# Genome-wide deficiency screen for the genomic regions responsible for heat resistance in *Drosophila melanogaster*

**DOI:** 10.1186/1471-2156-12-57

**Published:** 2011-06-22

**Authors:** Kazuo H Takahashi, Yasukazu Okada, Kouhei Teramura

**Affiliations:** 1 Research Core for Interdisciplinary Sciences, Okayama University, 3-1-1, Tsushima-naka, Kita-ku, Okayama 700-8530, Japan; 2 Graduate School of Environmental Science, Okayama University, 1-1-1, Tsushima-naka, Kita-ku, Okayama 700-8530, Japan

## Abstract

**Background:**

Temperature adaptation is one of the most important determinants of distribution and population size of organisms in nature. Recently, quantitative trait loci (QTL) mapping and gene expression profiling approaches have been used for detecting candidate genes for heat resistance. However, the resolution of QTL mapping is not high enough to examine the individual effects of various genes in each QTL. Heat stress-responsive genes, characterized by gene expression profiling studies, are not necessarily responsible for heat resistance. Some of these genes may be regulated in association with the heat stress response of other genes.

**Results:**

To evaluate which heat-responsive genes are potential candidates for heat resistance with higher resolution than previous QTL mapping studies, we performed genome-wide deficiency screen for QTL for heat resistance. We screened 439 isogenic deficiency strains from the DrosDel project, covering 65.6% of the *Drosophila melanogaster *genome in order to map QTL for thermal resistance. As a result, we found 19 QTL for heat resistance, including 3 novel QTL outside the QTL found in previous studies.

**Conclusion:**

The QTL found in this study encompassed 19 heat-responsive genes found in the previous gene expression profiling studies, suggesting that they were strong candidates for heat resistance. This result provides new insights into the genetic architecture of heat resistance. It also emphasizes the advantages of genome-wide deficiency screen using isogenic deficiency libraries.

## Background

Temperature adaptation is one of the most important determinants of the distribution and population size of organisms in nature. Fruit flies belonging to the genus *Drosophila *are a model system well suited for the study of thermal adaptation in natural and laboratory conditions. *Drosophila *populations from different geographical locations generally show different thermal resistances [1], indicating local temperature adaptation to the environment. In fact, Umina *et al*. [2] observed a shift in latitudinal cline in genetic polymorphisms in *Drosophila melanogaster *over 20 years and suggested that these findings were indicative of a rapid adaption to the increasingly warmer and drier climatic conditions. Balanya *et al*. [3] also observed a rapid response in the genetic polymorphisms of the invasive species *Drosophila subobscura *to various climatic conditions along the west coast of Chile and North America and suggested that this type of genetic polymorphism can be a useful tool to monitor the impact of global warming on the genetic structure of natural populations. Although the genetic machineries underlying such changes are still not completely understood, heat shock protein genes and other stress protein genes have been identified as potential candidates for heat resistance [1,4]. In particular, molecular chaperones such as *Hsp68 *[5] and *Hsp70 *[6] may contribute to heat resistance. However, the expression profiles of the known molecular chaperones do not always match the pattern of heat resistance [7,8], indicating the existence of unknown candidate genes.

Recently, QTL mapping and gene expression profiling approaches have been used to detect candidate genes for heat resistance. Norry *et al*. [9] searched for QTL that influenced knockdown resistance to high temperature (KRHT), mapped QTL for KRHT in 2 major autosomes of *D. melanogaster*, and examined the *X*-linked effects on KRHT. They found 4 QTL for KRHT, with 1 on the 2nd chromosome and the other 3 on the 3rd chromosome. They detected a significant contribution of the *X *chromosome to KRHT. The result of the abovementioned study suggests that QTL for heat resistance are not limited to the 3rd chromosome, where a number of heat shock protein genes are located, and that genes on other chromosomes may make a substantial contribution. After the initial study by Norry et al. [9], Morgan & Mackay [10] and Norry *et al*. [11] performed further QTL mapping and found several QTL that partially overlap with those found by Norry *et al*. [9]. Gene expression profiling after heat shock was performed at the embryonic stage by Leemans *et al*. [12] and at the adult stage by Sorensen *et al*. [13]. Leemans *et al*. [12] found that 74 of 1519 identified genes changed their relative expression in response to heat shock in the embryos of *D. melanogaster*. Among them, genes encoding heat shock proteins such as *Hsp22*, *Hsp26*, *Hsp27*, *Hsp23*, *Hsp67Bc*, *Hsp83*, *Hsp70Aa*, and *Hsp70Bb *were strongly upregulated (>3-fold), while 26 non-*Hsp *genes were also significantly upregulated by the heat shock [12]. Sorensen *et al*. [13] performed an analysis of heat stress response in *D. melanogaster *using whole genome gene expression arrays. They detected 1222 differentially expressed genes between the control and heat-selected strains and a variety of genes were included in the list of heat stress-responsive genes.

Although the attempts to detect candidate genes for heat resistance have significantly contributed to improving the understanding of the thermal resistance of organisms, we still need to narrow down the candidates in order to understand the complete picture of thermal resistance. The resolution of QTL mapping performed until now has not been high enough to examine the effect of individual genes in each QTL. Heat stress-responsive genes characterized by gene expression profiling studies are not necessarily the genes responsible for heat resistance; some may be regulated in association with the heat stress response of other genes. In addition, a common limit of the previous QTL mapping and gene expression profiling approaches is that detection of a certain QTL or gene with a significant effect depends on whether it is polymorphic between the 2 lines with different heat resistances used for mapping or comparison of the gene expression profile.

To cope with these problems, we performed genome-wide deficiency screen for QTL for heat resistance. A collection of isogenic deficiency strains provided by the DrosDel project [14,15] has a wide coverage over the *D. melanogaster *genome and has been used as an ideal tool for high resolution deficiency screen for polygenic traits [16,17]. Thus, QTL for heat resistance can be located precisely on the genome at a single base-pair resolution, and it is free from the abovementioned polymorphism issue because it can target arbitrary genome regions by design. In this study, we screened 439 deficiency strains covering 65.6% of the *D. melanogaster *genome to map QTL for thermal resistance. As a result, we reduced the large QTL found by Norry *et al*. (2004) to 16 much smaller candidate regions. We also discovered 3 novel QTL for heat resistance. The present study has shown that genome-wide deficiency screen is a powerful approach and may lead to a better understanding of heat resistance.

## Methods

### Flies

DrosDel isogenic deficiency strains were obtained from the Drosophila Genetic Resource Center (DGRC) in Kyoto, Japan, and were tested for heat resistance. The deficiency strains were constructed with a RS element-FLP system so that the breakpoints of the deletions can be determined at a single base-pair resolution [14]. The control strain DSK001 had an isogenic background with the deficiency strains, except for deletions, and a comparison of the survival of the control and deficiency strains would be an ideal approach for screening genome regions that are responsible for heat resistance. In this study, we used 439 DrosDel deficiency strains that cover about 65.6% of the whole genome region (Additional file 1, Table S1) out of 486 deficiency strains maintained in DGRC. The deficiency strains were selected to avoid the redundant deficiencies to perform efficient screen. Details of the deletion strains are available at the DrosDel web page http://www.drosdel.org.uk/.

### Experimental conditions

We screened for genome regions that affect heat resistance. Because most of the deficiencies used in this study are homozygous lethal, deficiency-control heterozygotes (Df/+) were tested for heat resistance. We collected 100 eggs from each of the crosses between the control and deletion strains, and then introduced them into a glass vial with fly medium. In a preliminary experiment, we checked that the egg density, 100 eggs per vial, was low enough not to cause intraspecific larval competition. For the deficiency strains that have a deletion on autosomes, we crossed the females of the control strain to males of each deficiency strain to control the maternal effect. Because deletions on the *X *chromosome in general result in lethality in the males, we crossed the males of the control strain to the females of each deficiency strain. One liter of fly medium (1000 mL water, 35 g dried yeast, 20 g soy flour, 73 g cornmeal, 30 g agar, 46.25 g malt extract, and 75 g dextrose) was mixed and boiled well. Then, 13.75 mL of an acid mix (412-mL propionic acid and 42-mL orthophosphoric acid made up to 1-L water) was mixed with 16.5 mL of nipagin (100-g methyl-p-hydroxybenzoate in 1-L 90% ethanol). Eggs were reared in incubators (MIR-254 or MIR-154, SANYO, Osaka, Japan) at 23°C or 28°C under constant light conditions. The temperature was double-checked daily with glass thermometers placed inside of the incubators. The laboratory where those incubators were located was air-conditioned, and the room temperature was maintained at around 23°C throughout the experiments. To keep the humidity level inside of the incubators constant, we placed a jug of water in the incubators. Emerging adults were sorted (target genotype: Df/+ and non-target genotype: balancer/+), and the number of females and males was recorded every 24 h. Five replicate vials were set up for each deletion strain at each temperature. To obtain control individuals (+/+), we collected 100 eggs from DSK001 and reared them as described above. The whole process of the egg introduction and adult sampling was done in series, and took about a year.

### Evaluation of heat sensitivity

Heat sensitivity of each deficiency strain was assessed by comparing the survival from the embryonic to adult stages between 23°C and 28°C thermal conditions. In nature, *Drosophila *larvae inhabit necrotic fruit where temperature may rise sufficiently to kill the larvae [18,19]. Growth, reproduction and other physiological processes work best at approximately 27°C or slightly below, and these functions deteriorate at higher temperatures. In fact, Krebs and Feder [18] observed natural genetic variation in larval heat resistance, and the trade-off between larval heat resistance and survival in a benign environment, indicating larval heat resistance is an ecologically important trait. For *D. melanogaster*, 23°C is a mild condition while 28°C is stressful condition when applied to the entire developmental period. In the previous studies, heat stress has been applied in various ways: 36°C for 1 h at adult stage in Sorensen et al. [13], 36°C for 25 minutes at embryonic stage in Leemans et al. [12], 38°C at adult stage in Morgan & Mackay [10]. Compared to those studies, the heat stress that we applied in the current study was milder in terms of the temperature, but was more severe in terms of the duration. The heat sensitivity has been evaluated in two ways: percent survival after heat stress or KRHT [9-11]. In the current study, the heat sensitivity was evaluated based on the survival because of the ease of scoring and statistical analyses. Based on our preliminary experiments, we expected that a large number of deficiency heterozygotes would show extremely low survival at higher temperature than 28°C when applied during the entire developmental period, making the statistical analysis difficult. The results from the previous studies and the current study are not directly comparable, but some genes may be associated with resistance to various heat stresses. *Hsp70 *genes, for example, are known to respond to both mild (25°C and 28°C) and severe (36°C and 38°C) heat stresses [12,13,20] in *D. melanogaster*. QTL with such broad temperature range in stress response would be detectable both in the current and the previous studies.

**Figure 1:**
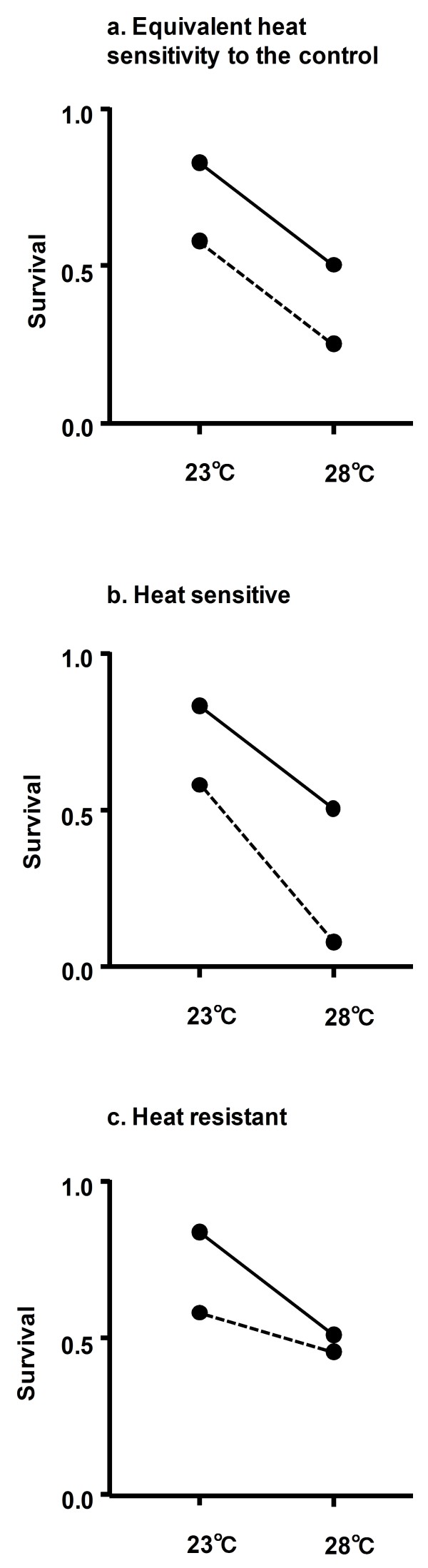
**The pattern of survival at 23°C and 28°C and the evaluation of heat sensitivity**. A solid line represents the change of survival between 23°C and 28°C for the control (+/+) and a broken line represents the change of survival between 23°C and 28°C for the deficiency heterozygote (Df/+). (a) Equivalent heat sensitivity between the control and deficiency heterozygote is shown. (b) A more heat sensitive deficiency heterozygote compared to the control is shown. (c) A more heat resistant deficiency heterozygote than the control is shown.

We paired the data from +/+ and each Df/+ and developed a generalized linear model (GLM) with a logit link function and binomial distribution to assess the heat sensitivity of each deficiency. We used the number of surviving and dead individuals as dependent variables and the genotype (+/+ or Df/+), temperature (23°C or 28°C), and their interaction as independent variables. The heat sensitivity was evaluated based on the sign and significance of the coefficient of the interaction term. Survival of the control strain DSK001 was reduced at 28°C compared to that at 23°C. If the paired deficiency strain had the same reduction in survival with the increased temperature as did the control, the coefficient of the interaction term would be zero, indicating no change in the heat sensitivity (Figure 1a). If the paired deficiency strain had a significantly greater reduction in survival with the increased temperature than that of the control strain, the coefficient of the interaction term would result in a positive value (Figure 1b). This indicates that the Df/+ individuals are more heat sensitive than +/+ individuals or, in other words, the genes encompassed by the deletion contribute to heat resistance. If the paired deficiency strain showed a significantly smaller reduction in survival (or increase of survival) with increased temperature, the coefficient of the interaction term would be a negative value, indicating higher heat resistance compared to that of the control strain (Figure 1c). Because we developed 439 GLMs for each sex in order to evaluate the heat sensitivity of each deficiency, we had a multiple comparisons problem in the evaluation of the significance of the interaction term between genotype and temperature. In the current study, instead of controlling the familywise error rate, we controlled the expected proportion of falsely rejected hypotheses-the false discovery rate (FDR) to ensure statistical power under a large number of multiple tests. This error rate is equivalent to the familywise error rate when all hypotheses are true but is smaller otherwise [21]. To adjust for this issue, we applied a Benjamini-Hochberg procedure to control the FDR [21]. Genomic regions encompassed by deficiencies were defined as heat resistance QTL if they had positive coefficients of the interaction term with adjusted FDR *p*-values (i.e., the *q*-value) smaller than 0.05 for the coefficient. All the analyses were conducted with statistical software R (version 2.8.1).

## Results

**Figure 2:**
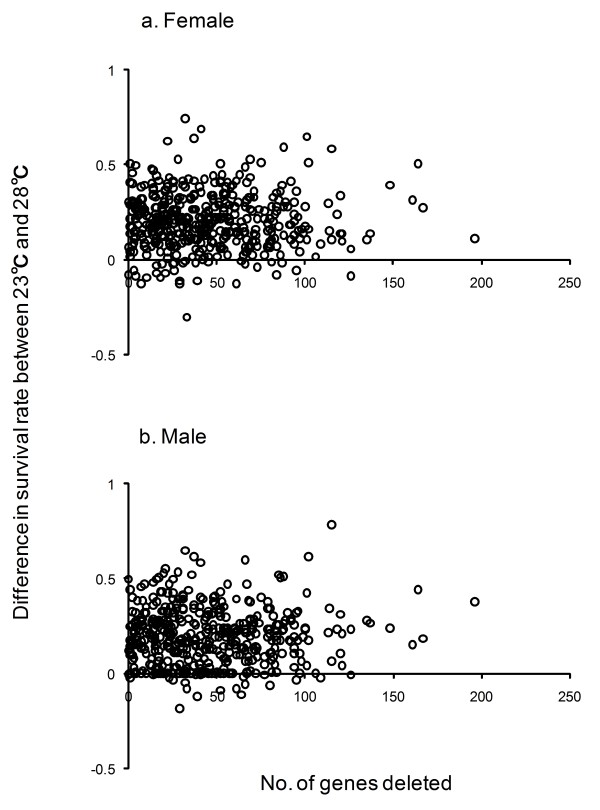
**The correlation between the number of genes deleted in each deficiency and the difference in the survival rate of the deficiency heterozygote at 23°C and 28°C for females (a) and males (b)**.

Because each deficiency encompasses a different number of genes (0 to 196), we checked whether the deletion of a number of genes in general influenced the heat resistance. The correlation between the number of deleted genes and the difference in the mean survival rates at 23°C and 28°C of each deficiency strain was very weak and insignificant in both females (correlation coefficient: 0.005, *P *> 0.91) and males (correlation coefficient: 0.019, *P *> 0.68) (Figure 2). The difference was larger than zero in 406 deficiencies in females and 415 in males, indicating that the survival rate was higher at 23°C than at 28°C (Figure 2).

**Figure 3:**
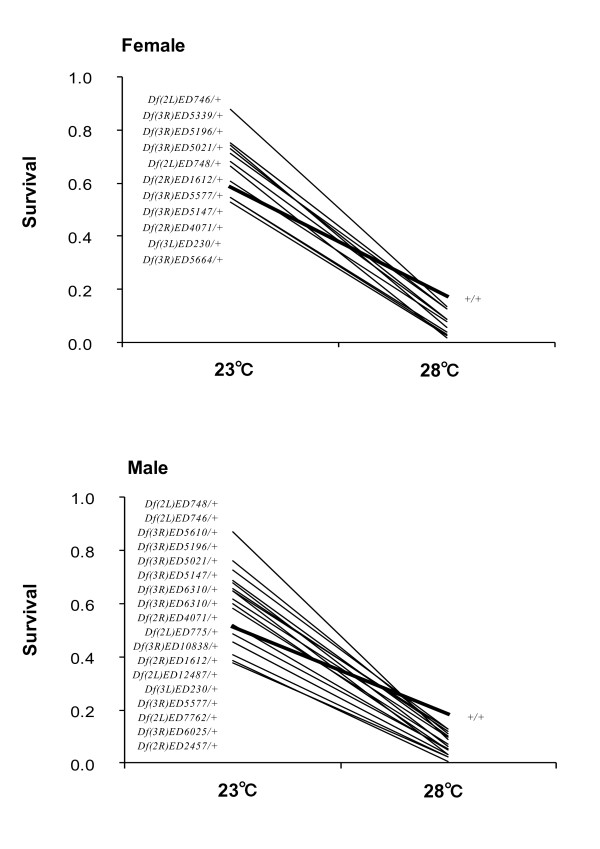
**The mean survival of the deficiency heterozygotes (Df/+) with significant heat sensitivity (thin lines) and the control (+/+) (broad lines) at 23°C and 28°C for females (a) and males (b)**.

**Figure 4:**
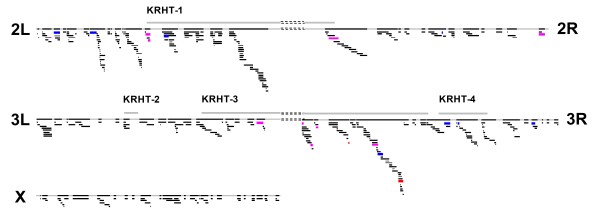
**Distribution of deficiencies on the 2nd, 3rd, and *X *chromosomes**. Genome regions covered by deficiencies are filled with black and bars below each chromosome represent the location of each deficiency. Bars for deficiencies with significant heat sensitivity are filled with different colors based on sex-specificity (a significant effect only in females is in red, a significant effect only in males is in blue, and a significant effect in both females and males is in purple).

Among 439 deficiencies that we examined, 19 deficiencies showed a positive and significant coefficient of the interaction term between genotype (+/+ or Df/+) and temperature (23°C or 28°C), indicating their significant effect to enhance heat sensitivity (Figure 3). The 19 deficiencies with significant heat sensitivities were distributed over the 2nd and 3rd chromosomes, but not over the *X *chromosome (Figure 4). On the 2nd chromosome, the deficiencies with significant effect to enhance heat sensitivity were scattered on both chromosome arms and 8 of them were centered around the centromere on the 3rd chromosome (Figure 4). Eight of the 19 deficiencies had a significant effect in females and males and 11 of them had a sex-specific effect (Figure 4 and Table 1). Some deficiencies with significant effect to enhance heat sensitivity had a higher mean survival at 23°C than the controls (+/+) but all of them showed a lower survival than the controls at 28°C. In total, 1037 genes were encompassed by these deletions with significant effect to enhance heat sensitivity. The 3 QTL for KRHT found by Norry *et al*. [9] (KRHT-1, KRHT-3, and KRHT-4) encompassed 16 of the 19 deficiencies (Table 1). QTL found by Morgan & Mackay [10] and Norry *et al*. [11] encompassed 4 deficiencies with significant effect to enhance heat sensitivity found in the current study (Table 1). Among them, 3 deficiencies with significant effect to enhance heat sensitivity encompassed the heat-responsive genes found by Leemans *et al*. [12] while 11 deficiencies encompassed the non-overlapping heat-responsive genes found by Sorensen *et al*. [13] (Table 1).

**Table 1:** Deficiencies that showed significant heat sensitivity, corresponding QTL from Norry *et al*. (2004), Morgan & Mackay (2006) and Norry *et al*. (2008), and stress resonsive genes from Leemans *et al*. (2000) and Sorensen *et al*. (2005).

Chromosome	Deletion ID	Region	No. of genes deleted	Sex-specificity	QTL from Norry *et al*. (2004)	QTL from Morgan. & Mackay (2006)	QTL from Norry et al (2008)	Heat responsive genes from Leemans *et al*. (2000)	Heat responsive genes from Sorensen *et al*. (2005)
2L	*Df(2L)ED746*	31F4 - 32A5	34	Female & male	KRHT-1				CG7300, CG17124
	*Df(2L)ED748*	31B1 - 32A5	116	Female & male	KRHT-1				CG5390, CG17108
	*Df(2L)ED775*	33B8 - 34A3	80	Male	KRHT-1				
	*Df(2L)ED7762*	22A6 - 22D3	70	Male	KRHT-1				*Cyp4ac2*
	*Df(2L)ED12487*	25C3 - 25F2	70	Male	KRHT-1		KRHT		CG3270
2R	*Df(2R)ED1612*	42A13 - 42E6	87	Female & male	KRHT-1	H3	KRHT		*Ance-5*
	*Df(2R)ED2457*	52D11 - 52E7	24	Male					
	*Df(2R)ED4071*	60C8 - 60E8	101	Female & male					
3L	*Df(3L)ED230*	79C2 - 80A4	51	Female & male	KRHT-3			*Ten-m*	
3R	*Df(3R)ED5021*	82A1 - 82B1	35	Female & male	KRHT-3				
	*Df(3R)ED5147*	82E8 - 83A1	27	Female & male	KRHT-3				
	*Df(3R)ED5196*	83B9 - 83D2	39	Female & male	KRHT-3				
	*Df(3R)ED5339*	85D1 - 85D11	21	Female	KRHT-3				CG16749
	*Df(3R)ED5577*	86F9 - 87B13	114	Female & male	KRHT-3	H4		*Hsp70Ab*	*GstD2*, *GstD5*, CG32919
	*Df(3R)ED5610*	87B11 - 87D7	63	Male	KRHT-3	H4		*Hsp70Bb*	*Hsp70Bbb *, *Hsp70Bc*
	*Df(3R)ED5664*	88D1 - 88E3	54	Female	KRHT-3	H4			CG3984
	*Df(3R)ED6025*	92A11 - 92E2	76	Male	KRHT-4		KRHT		CG5023, CG3301
	*Df(3R)ED6310*	98F12 - 99B2	36	Male					
	*Df(3R)ED10838*	93C1 - 93D4	21	Male	KRHT-4		KRHT		

## Discussion

In this study, we conducted a genome-wide deficiency screen for QTL for heat resistance. The correlation between the number of deleted genes and the degree of heat sensitivity was not significant, suggesting that the deletion of a number of genes in general did not influence heat sensitivity. As a result, we found 19 QTL for heat resistance on the 2nd and 3rd chromosomes. The result of the present study corresponded well to QTL mapping study conducted by Norry *et al*. [9] because 16 QTL found in this study were encompassed by QTL for KRHT found by Norry *et al*. [9]. Norry *et al*. [22] localized 5 QTL for KRHT, and 3 of them encompassed 4 of the QTL for heat resistance found in this study. Similar distribution of QTL for heat resistance, i.e., resistance to mild heat stress during the pre-adult period, and those for KRHT suggest that the underlying mechanisms for those traits may partially overlap with each other, although they contribute to different aspects of heat resistance. In addition, colocalization of 4 QTL found in this study with 2 QTL found by Morgan & Mackay [10] suggests that resistance to mild heat stress during the pre-adult period and short-term heat shock at the adult stage share genetic architecture. Three of the heat-responsive genes found by Leemans *et al*. [12] were located in QTL found in this study, while the 16 non-overlapping heat-responsive genes found by Sorensen *et al*. [13] were encompassed by 11 QTL found in this study. To confirm whether QTL found in the previous studies and the ones found in the current study consist of the same genes, complementation test using both the deficiency strains and the strains from the previous QTL mapping studies would be necessary in future studies. Part of the reason for the difference could be the method of heat stress application. Leemans *et al*. [12] applied a short heat shock at the embryonic stage, while we provided mild heat stress (28°C) during the entire pre-adult stages. In addition, the previous QTL mapping studies utilized natural genetic variation to map candidate QTL while we used deficiency screening targeting arbitrary genome regions by design. When natural genetic variation is utilized to map QTL, the effect of the loci would not be detected if there is no genetic variation in the focal loci between the strains used for the mapping. Because QTL mapping is usually done with two representative strains, variable regions on the genome is limited. On the other hand, when well-designed deficiency collections such as DrosDel isogenic deficiency collections were used to map QTL, genetic variation between the deficiency and the control strains can be located even where no natural genetic variation exists in wild populations. The difference in the sources of genetic variation for the mappings could cause the difference of the QTL found in the previous and current studies.

Among the heat-responsive genes from Leemans *et al*. [12] and Sorensen *et al*. [13], 4 *Hsp *genes, *Hsp70Ab*, *Hsp70Bb*, *Hsp70Bbb*, and *Hsp70Bc*, were located in the QTL found in this study. In addition, 2 *Hsp *genes, *Hsp70Aa and Hsp70Ba*, were included in the QTL of which locations corresponded to the deficiency region of *Df(3R)ED5577*. These 6 copies of *Hsp70 *are nearly identical in sequence and are closely linked together on 87A and 87C of the right arm of the 3rd chromosome [23]. Rapid induction of *Hsp70 *expression upon heat shock and the suppression of its expression are strictly regulated in a cell [24,25]. The effect of *Hsp70*s on heat resistance has also been confirmed at the individual level based on the increased heat resistance in organisms with a high copy number of *Hsp70 *genes [26] and on the reduced heat resistance in *Hsp70-*null flies [27]. We detected the effect of QTL, including those genes in our screening, and the findings support that *Hsp70 *genes are strong candidates for heat resistance in *D. melanogaster*. It also indicates that our screening has sufficient detection power to confirm the effect of *Hsp70 *genes and will be able to detect QTL with equivalent contribution to heat resistance as *Hsp70 *genes.

Six of the non-*Hsp *heat-responsive genes from Sorensen *et al*. [13] that were located inside QTL found in this study, CG17124, CG17108, CG3270, *GstD2*, *GstD5*, CG3301, have been reported to be involved in various stress responses, such as starvation stress, aging, and oxidative stress, and pesticide resistance [28-32]. There were 2 genes that were suggested to be involved in immune response: CG16749 and *GstD5 *[33,34]. A general responsiveness to environmental stress of these genes suggests that they are strong candidates for heat resistance. Testing the individual effect of those genes on heat resistance will be necessary to understand how *Hsp *and non-*Hsp *genes act together to contribute to heat resistance. In addition, this result may suggest that the resistance for mild heat stress (28°C) during the entire pre-adult stage consists of multiple stress-resistance processes that are required for comprehensive homeostasis of development. Compared to a short-term heat shock at embryonic or adult stages, examination of the response to a long-term heat stress during the pre-adult stage may reveal ecologically important mechanisms for heat resistance in natural conditions.

In our deficiency screen, 3 QTL were located outside the QTL found in the previous QTL mapping studies [9-11]. As described above, the difference in heat stress application between the present study and others may result in this difference. The novel QTL may contain genes that specifically contribute to long-term mild heat stress response during the pre-adult period. One QTL corresponded to the deficiency region of *Df(2R)ED4071 *contained a heat-responsive gene, *Ance-5*, but the other two corresponded to the deficiency regions of *Df(2R)ED2457 *and *Df(3R)ED6310 *did not include any known heat-responsive genes. The 2 QTL contained 24 and 36 genes. A detailed examination of those genes in future research may reveal novel candidate genes for heat resistance.

The *X*-linked effect observed by Norry *et al*. [9] and Norry *et al*. [22] was not detected in this study. Part of the reason may be that most deficiencies on the *X *chromosome result in lethality in the male (see Additional file 1, Table S1). Thus, we evaluated heat sensitivity for the *X *chromosome in many cases for females only in this study, while Norry *et al*. [9] and Norry *et al*. [22] only used male flies for their QTL mapping. In addition, coverage of deficiencies was lowest on the *X *chromosome (54.1%) in this study, and it may lower the detection power on this chromosome. An effort to map with a higher coverage using both sexes is necessary for a more detailed analysis of the *X *chromosome.

In the current study, we searched for genomic deletions that increased the heat sensitivity to locate genomic regions with effect on heat tolerance, but we also found genomic deletions that decreased heat sensitivity. Such unexpected effect of the deletions could be due to the enhanced heat resistance at larval stage in exchange for fitness at adult stage. In this study, unfortunately, we did not measure their fitness at adult stage, and whether such trade-off exists or not is still unknown. Decreased heat sensitivity of the deficiency heterozygotes indicates that those QTL function to suppress heat tolerance in DSK001 homozygotes. No genes or no QTL have been reported to suppress heat resistance so far. Further study is necessary to understand the mechanism of the regulation of heat sensitivity thoroughly.

QTL mapping has been the most popular method to map candidate genomic regions for quantitative traits. Due to the recent development of the isogenic deficiency libraries such as DrosDel and Exelixis collections [14,35], genome-wide deficiency screen became possible in *D. melanogaster*. In the current study, most of the deletions caused homozygous lethality, and it limits the experimental design to compare +/+ to Df/+. The large deletions suitable for an efficient screen tend to encompass recessive lethal alleles or alleles that cause recessive lethality when deleted together. In this experimental setting, QTL with dominant or recessive effect would not be detectable because +/+ and Df/+ are expected to show the same phenotype. In addition, when deficiencies with significant effect overlap with deficiencies without effect, it is possible to subtract the overlapping regions to reduce candidate regions down to smaller sizes. In the current study, however, there were cases where a deletion with significant effect was completely encompassed by a larger deletion without significant effect. Large deletions tend to encompass genes essential for survival, and it makes the subtraction approach lead to a false refinement of the candidate regions. To present the result in a conservative way, we avoided the subtraction approach in the current study. Even with this limitation, however, we detected 19 QTL for heat resistance, indicating that it is an effective approach when targeting QTL with additive effect. As for the resolution of the mapping, previously located QTL for heat resistance usually encompassed from several hundreds to thousands genes [9-11], while the QTL found in this study encompassed about 58 genes on average. The more than 10 times higher resolution of the present deficiency screen compared to the previous QTL mapping makes it possible to analyze the effect of individual genes in each QTL using mutation or RNAi approach in future studies. Although intensive effort is necessary to achieve high genome coverage, the current isogenic deficiency screen is a powerful approach to investigate genetic architecture of quantitative traits in *D. melanogaster*.

In the present study, we performed a novel screening for QTL for heat resistance and found 16 that overlapped with the previously known QTL. Combined with the results of gene expression profiling studies, we specified several putative candidate genes in the QTL. We also discovered 3 novel QTL for heat resistance. The high resolution mapping in this study compared to that of the previous QTL mapping studies has the advantage of a genome-wide screening using a newly available genetic tool, which is a collection of isogenic deficiency strains. Further deficiency screen with smaller deficiencies within the genomic regions with significant effect found in the current study would narrow down the candidate genes from the average 58 genes per candidate regions to smaller numbers. This makes the examination of the individual candidate genes using mutation analysis or RNAi knockdown more feasible in future studies. In addition to that, genome-wide deficiency screen of QTL for other aspects of heat resistance combined with more detailed gene expression profiling studies may provide a better understanding of the underlying mechanisms of heat resistance.

## Conclusions

We performed genome-wide deficiency screen of QTL for heat resistance with higher resolution than the previous QTL mappings, and found 19 QTL. Sixteen of the QTL partially overlapped with the previously found QTL while 3 of them were novel QTL for heat resistance. Our fine mapping reduced the number of candidate genes significantly, and provided new insights into the genetic architecture of heat resistance. This also emphasizes the advantage of the genome-wide deficiency screen using isogenic deficiency libraries.

## Authors' contributions

KHT designed the experiments. KHT, YO and KT conducted the experiments. KHT analyzed and interpreted the data and drafted the manuscript. All authors revised the manuscript critically for important intellectual content, participated in the discussions and approved its final form.

## Supplementary Material

Additional file 1 **The control and deficiencies used for the mapping, and their location, size, and mean survival rate from eggs to adults with standard deviation in parentheses**. The control and deficiencies used for the mapping, and their location, size, and mean survival rate from eggs to adults with standard deviation in parentheses. Click here for file
