# Supplementary material for: Genome-wide deficiency screen for the genomic regions responsible for heat resistance in Drosophila melanogaster
Source: BMC Genet. 2011 Jun 22;12:57. doi: 10.1186/1471-2156-12-57 (PMC3146426; doi:10.1186/1471-2156-12-57)
Supplement: Additional file 1 — The control and deficiencies used for the mapping, and their location, size, and mean survival rate from eggs to adults with standard deviation in parentheses. The control and deficiencies used for the mapping, and their location, size, and mean survival rate from eggs to adults with standard deviation in parentheses. [file 1471-2156-12-57-S1.PDF]

Table S1. The control and deficiencies used for the mapping, and their location, size, and mean survival rate from eggs to adults with standard deviation in parentheses.

| Chromosome | Deficiency strain | Region | Deletion size (bp) | Female | | Male | |
|---|---|---|---|---|---|---|---|
| | | | | 23°C | 28°C | 23°C | 28°C |
| - | DSK001 | - | 0 | 0.58 ( 0.15 ) | 0.18 ( 0.06 ) | 0.51 ( 0.18 ) | 0.18 ( 0.12 ) |
| 2L | Df(2L)ED3 | 35B2 - 35D1 | 843185 | 0.44 ( 0.23 ) | 0.18 ( 0.11 ) | 0.31 ( 0.24 ) | 0.26 ( 0.20 ) |
| | Df(2L)ED21 | 21B3 - 21B7 | 125158 | 0.36 ( 0.19 ) | 0.17 ( 0.05 ) | 0.62 ( 0.20 ) | 0.12 ( 0.10 ) |
| | Df(2L)ED40 | 21D1 - 21D2 | 9980 | 0.58 ( 0.13 ) | 0.51 ( 0.09 ) | 0.52 ( 0.12 ) | 0.53 ( 0.08 ) |
| | Df(2L)ED49 | 1A1 - 100E1 | 19888 | 0.58 ( 0.18 ) | 0.26 ( 0.16 ) | 0.60 ( 0.19 ) | 0.46 ( 0.20 ) |
| | Df(2L)ED87 | 21E2 - 21E2 | 284732 | 0.58 ( 0.14 ) | 0.46 ( 0.22 ) | 0.53 ( 0.20 ) | 0.37 ( 0.08 ) |
| | Df(2L)ED94 | 21E2 - 21E3 | 468874 | 0.53 ( 0.21 ) | 0.12 ( 0.05 ) | 0.47 ( 0.21 ) | 0.06 ( 0.05 ) |
| | Df(2L)ED105 | 21E2 - 22A1 | 567674 | 0.38 ( 0.10 ) | 0.31 ( 0.17 ) | 0.37 ( 0.11 ) | 0.25 ( 0.10 ) |
| | Df(2L)ED108 | 21F1 - 22A1 | 301394 | 0.79 ( 0.18 ) | 0.43 ( 0.13 ) | 0.02 ( 0.04 ) | 0.00 ( 0.00 ) |
| | Df(2L)ED122 | 22B1 - 22D4 | 494297 | 0.37 ( 0.15 ) | 0.10 ( 0.04 ) | 0.38 ( 0.13 ) | 0.10 ( 0.05 ) |
| | Df(2L)ED123 | 22B8 - 22D4 | 236161 | 0.49 ( 0.25 ) | 0.43 ( 0.12 ) | 0.47 ( 0.09 ) | 0.33 ( 0.15 ) |
| | Df(2L)ED124 | 22D3 - 22D4 | 23445 | 0.54 ( 0.06 ) | 0.28 ( 0.14 ) | 0.48 ( 0.12 ) | 0.23 ( 0.12 ) |
| | Df(2L)ED125 | 22B2 - 22D4 | 484626 | 0.36 ( 0.12 ) | 0.19 ( 0.07 ) | 0.46 ( 0.15 ) | 0.19 ( 0.10 ) |
| | Df(2L)ED132 | 23A3 - 23A3 | 106 | 0.63 ( 0.14 ) | 0.71 ( 0.15 ) | 0.63 ( 0.13 ) | 0.51 ( 0.14 ) |
| | Df(2L)ED136 | 22F4 - 23A3 | 260190 | 0.69 ( 0.26 ) | 0.38 ( 0.10 ) | 0.51 ( 0.19 ) | 0.35 ( 0.15 ) |
| | Df(2L)ED206 | 23B8 - 23C5 | 181763 | 0.65 ( 0.29 ) | 0.43 ( 0.15 ) | 0.62 ( 0.10 ) | 0.34 ( 0.10 ) |
| | Df(2L)ED216 | 23B8 - 23C5 | 181892 | 0.64 ( 0.06 ) | 0.22 ( 0.07 ) | 0.67 ( 0.08 ) | 0.23 ( 0.06 ) |
| | Df(2L)ED234 | 23C4 - 24A2 | 632936 | 0.36 ( 0.30 ) | 0.44 ( 0.20 ) | 0.41 ( 0.24 ) | 0.40 ( 0.10 ) |
| | Df(2L)ED243 | 24A2 - 24A4 | 24683 | 0.58 ( 0.08 ) | 0.36 ( 0.08 ) | 0.51 ( 0.16 ) | 0.37 ( 0.21 ) |
| | Df(2L)ED247 | 24A2 - 24C3 | 138959 | 0.38 ( 0.14 ) | 0.02 ( 0.04 ) | 0.44 ( 0.12 ) | 0.09 ( 0.05 ) |
| | Df(2L)ED250 | 24F4 - 25A7 | 344209 | 0.80 ( 0.19 ) | 0.46 ( 0.23 ) | 0.77 ( 0.32 ) | 0.34 ( 0.15 ) |
| | Df(2L)ED256 | 25B1 - 25B10 | 108097 | 0.42 ( 0.17 ) | 0.29 ( 0.14 ) | 0.42 ( 0.25 ) | 0.31 ( 0.19 ) |
| | Df(2L)ED270 | 25F2 - 25F5 | 141567 | 0.40 ( 0.14 ) | 0.30 ( 0.13 ) | 0.55 ( 0.13 ) | 0.28 ( 0.08 ) |
| | Df(2L)ED279 | 25F2 - 26A1 | 248827 | 0.46 ( 0.12 ) | 0.19 ( 0.09 ) | 0.56 ( 0.23 ) | 0.22 ( 0.11 ) |
| | Df(2L)ED280 | 25F5 - 26A1 | 105526 | 0.76 ( 0.17 ) | 0.47 ( 0.24 ) | 0.52 ( 0.06 ) | 0.34 ( 0.15 ) |
| | Df(2L)ED284 | 25F2 - 26A3 | 285333 | 0.74 ( 0.18 ) | 0.35 ( 0.02 ) | 0.64 ( 0.14 ) | 0.23 ( 0.11 ) |
| | Df(2L)ED285 | 25F5 - 26A3 | 142032 | 0.74 ( 0.13 ) | 0.30 ( 0.17 ) | 0.66 ( 0.10 ) | 0.28 ( 0.15 ) |
| | Df(2L)ED292 | 25F5 - 26B2 | 179079 | 0.40 ( 0.19 ) | 0.14 ( 0.14 ) | 0.47 ( 0.14 ) | 0.10 ( 0.05 ) |
| | Df(2L)ED299 | 26B1 - 26B2 | 2194 | 0.24 ( 0.18 ) | 0.07 ( 0.05 ) | 0.20 ( 0.20 ) | 0.03 ( 0.03 ) |
| | Df(2L)ED330 | 26A3 - 26B2 | 55750 | 0.35 ( 0.13 ) | 0.18 ( 0.11 ) | 0.42 ( 0.11 ) | 0.23 ( 0.18 ) |
| | Df(2L)ED331 | 26B2 - 26B2 | 18588 | 0.84 ( 0.26 ) | 0.70 ( 0.14 ) | 0.01 ( 0.02 ) | 0.01 ( 0.02 ) |
| | Df(2L)ED334 | 25F2 - 26B2 | 341038 | 0.38 ( 0.18 ) | 0.20 ( 0.15 ) | 0.43 ( 0.21 ) | 0.10 ( 0.06 ) |
| | Df(2L)ED343 | 26B2 - 26B5 | 82250 | 0.49 ( 0.16 ) | 0.11 ( 0.11 ) | 0.62 ( 0.30 ) | 0.20 ( 0.15 ) |
| | Df(2L)ED347 | 25F5 - 26B5 | 280456 | 0.40 ( 0.16 ) | 0.26 ( 0.11 ) | 0.42 ( 0.14 ) | 0.19 ( 0.15 ) |
| | Df(2L)ED353 | 26B2 - 26B5 | 83109 | 0.38 ( 0.14 ) | 0.40 ( 0.18 ) | 0.29 ( 0.24 ) | 0.32 ( 0.20 ) |
| | Df(2L)ED354 | 26B1 - 26B5 | 102961 | 0.06 ( 0.06 ) | 0.18 ( 0.20 ) | 0.05 ( 0.07 ) | 0.23 ( 0.15 ) |
| | Df(2L)ED369 | 26C3 - 26D1 | 72246 | 0.67 ( 0.17 ) | 0.19 ( 0.10 ) | 0.55 ( 0.16 ) | 0.28 ( 0.15 ) |
| | Df(2L)ED371 | 26C3 - 26D1 | 73530 | 0.60 ( 0.17 ) | 0.22 ( 0.16 ) | 0.70 ( 0.13 ) | 0.22 ( 0.08 ) |
| | Df(2L)ED373 | 26B2 - 26D1 | 430254 | 0.06 ( 0.02 ) | 0.18 ( 0.08 ) | 0.07 ( 0.08 ) | 0.15 ( 0.13 ) |
| | Df(2L)ED374 | 26B10 - 26D1 | 232319 | 0.62 ( 0.23 ) | 0.41 ( 0.09 ) | 0.00 ( 0.01 ) | 0.00 ( 0.00 ) |
| | Df(2L)ED384 | 26B2 - 26D7 | 465648 | 0.22 ( 0.28 ) | 0.08 ( 0.07 ) | 0.16 ( 0.23 ) | 0.02 ( 0.04 ) |
| | Df(2L)ED385 | 26B1 - 26D7 | 485500 | 0.66 ( 0.15 ) | 0.23 ( 0.12 ) | 0.47 ( 0.08 ) | 0.19 ( 0.09 ) |
| | Df(2L)ED438 | 27D1 - 27D4 | 52278 | 0.62 ( 0.26 ) | 0.36 ( 0.12 ) | 0.58 ( 0.10 ) | 0.38 ( 0.25 ) |
| | Df(2L)ED440 | 27D3 - 27E1 | 74563 | 0.28 ( 0.10 ) | 0.11 ( 0.05 ) | 0.28 ( 0.16 ) | 0.16 ( 0.10 ) |
| | Df(2L)ED463 | 27F4 - 27F7 | 661 | 0.78 ( 0.17 ) | 0.48 ( 0.21 ) | 0.77 ( 0.19 ) | 0.27 ( 0.09 ) |
| | Df(2L)ED478 | 27F7 - 28B1 | 139196 | 0.72 ( 0.19 ) | 0.58 ( 0.27 ) | 0.77 ( 0.22 ) | 0.54 ( 0.09 ) |
| | Df(2L)ED494 | 27F4 - 28B1 | 153371 | 0.58 ( 0.13 ) | 0.25 ( 0.10 ) | 0.50 ( 0.20 ) | 0.32 ( 0.14 ) |
| | Df(2L)ED496 | 28C4 - 28C4 | 9590 | 0.84 ( 0.17 ) | 0.54 ( 0.19 ) | 0.74 ( 0.29 ) | 0.58 ( 0.25 ) |
| | Df(2L)ED501 | 27F7 - 28C4 | 376256 | 0.59 ( 0.23 ) | 0.15 ( 0.11 ) | 0.57 ( 0.24 ) | 0.31 ( 0.30 ) |
| | Df(2L)ED502 | 28C1 - 28C4 | 122088 | 0.62 ( 0.15 ) | 0.55 ( 0.12 ) | 0.61 ( 0.22 ) | 0.46 ( 0.09 ) |
| | Df(2L)ED508 | 28B1 - 28C4 | 223552 | 0.17 ( 0.08 ) | 0.47 ( 0.21 ) | 0.26 ( 0.18 ) | 0.34 ( 0.21 ) |
| | Df(2L)ED517 | 27F7 - 28D2 | 447744 | 0.46 ( 0.08 ) | 0.07 ( 0.06 ) | 0.34 ( 0.12 ) | 0.18 ( 0.09 ) |
| | Df(2L)ED548 | 28E1 - 28E9 | 91467 | 0.72 ( 0.17 ) | 0.35 ( 0.19 ) | 0.65 ( 0.09 ) | 0.29 ( 0.15 ) |
| | Df(2L)ED573 | 28F1 - 29A2 | 95377 | 0.45 ( 0.10 ) | 0.52 ( 0.20 ) | 0.54 ( 0.16 ) | 0.38 ( 0.23 ) |
| | Df(2L)ED578 | 28F1 - 29A3 | 103066 | 0.38 ( 0.07 ) | 0.19 ( 0.15 ) | 0.41 ( 0.21 ) | 0.18 ( 0.05 ) |
| | Df(2L)ED611 | 29B4 - 29C3 | 36967 | 0.60 ( 0.16 ) | 0.38 ( 0.15 ) | 0.58 ( 0.14 ) | 0.30 ( 0.09 ) |
| | Df(2L)ED623 | 29C1 - 29E4 | 296560 | 0.60 ( 0.21 ) | 0.43 ( 0.18 ) | 0.56 ( 0.27 ) | 0.31 ( 0.11 ) |
| | Df(2L)ED629 | 29B4 - 29E4 | 317273 | 0.63 ( 0.10 ) | 0.42 ( 0.10 ) | 0.69 ( 0.23 ) | 0.58 ( 0.18 ) |
| | Df(2L)ED630 | 29C3 - 29E4 | 278827 | 0.46 ( 0.08 ) | 0.31 ( 0.10 ) | 0.35 ( 0.10 ) | 0.21 ( 0.09 ) |
| | Df(2L)ED632 | 29E1 - 29E4 | 156152 | 0.52 ( 0.24 ) | 0.30 ( 0.10 ) | 0.38 ( 0.13 ) | 0.17 ( 0.06 ) |
| | Df(2L)ED647 | 29E1 - 29F5 | 414176 | 0.38 ( 0.15 ) | 0.18 ( 0.12 ) | 0.37 ( 0.12 ) | 0.18 ( 0.07 ) |
| | Df(2L)ED659 | 29E1 - 30A3 | 646785 | 0.63 ( 0.14 ) | 0.39 ( 0.16 ) | 0.52 ( 0.07 ) | 0.37 ( 0.19 ) |
| | Df(2L)ED673 | 30A4 - 30B3 | 226380 | 0.49 ( 0.15 ) | 0.20 ( 0.06 ) | 0.46 ( 0.18 ) | 0.14 ( 0.10 ) |
| | Df(2L)ED677 | 30B3 - 30B12 | 144271 | 0.42 ( 0.17 ) | 0.33 ( 0.16 ) | 0.49 ( 0.27 ) | 0.32 ( 0.20 ) |
| | Df(2L)ED678 | 29F5 - 30B12 | 623585 | 0.58 ( 0.08 ) | 0.49 ( 0.19 ) | 0.65 ( 0.17 ) | 0.42 ( 0.13 ) |
| | Df(2L)ED679 | 30B12 - 30B12 | 10552 | 0.52 ( 0.15 ) | 0.26 ( 0.24 ) | 0.63 ( 0.11 ) | 0.37 ( 0.30 ) |
| | Df(2L)ED680 | 30A4 - 30B12 | 376664 | 0.56 ( 0.35 ) | 0.24 ( 0.07 ) | 0.41 ( 0.10 ) | 0.20 ( 0.07 ) |
| | Df(2L)ED684 | 30B12 - 30C1 | 42145 | 0.62 ( 0.16 ) | 0.30 ( 0.12 ) | 0.57 ( 0.05 ) | 0.18 ( 0.09 ) |
| | Df(2L)ED690 | 30B3 - 30E4 | 480705 | 0.26 ( 0.15 ) | 0.14 ( 0.05 ) | 0.23 ( 0.12 ) | 0.14 ( 0.07 ) |
| | Df(2L)ED692 | 30B12 - 30E4 | 346986 | 0.52 ( 0.22 ) | 0.11 ( 0.05 ) | 0.40 ( 0.20 ) | 0.10 ( 0.05 ) |
| | Df(2L)ED695 | 30C5 - 30E4 | 218967 | 0.66 ( 0.18 ) | 0.43 ( 0.09 ) | 0.76 ( 0.19 ) | 0.41 ( 0.16 ) |
| | Df(2L)ED697 | 30C1 - 30E4 | 301348 | 0.57 ( 0.15 ) | 0.29 ( 0.10 ) | 0.54 ( 0.05 ) | 0.41 ( 0.22 ) |
| | Df(2L)ED700 | 30E1 - 30E4 | 20668 | 0.58 ( 0.12 ) | 0.20 ( 0.12 ) | 0.58 ( 0.28 ) | 0.12 ( 0.09 ) |
| | Df(2L)ED701 | 30C5 - 30F1 | 249119 | 0.50 ( 0.24 ) | 0.19 ( 0.08 ) | 0.44 ( 0.23 ) | 0.10 ( 0.04 ) |
| | Df(2L)ED729 | 31B1 - 31D7 | 100900 | 0.45 ( 0.19 ) | 0.12 ( 0.04 ) | 0.30 ( 0.17 ) | 0.09 ( 0.07 ) |
| | Df(2L)ED746 | 31F4 - 32A5 | 225931 | 0.88 ( 0.20 ) | 0.14 ( 0.08 ) | 0.76 ( 0.19 ) | 0.11 ( 0.08 ) |
| | Df(2L)ED748 | 31B1 - 32A5 | 485690 | 0.71 ( 0.23 ) | 0.13 ( 0.09 ) | 0.87 ( 0.15 ) | 0.09 ( 0.04 ) |
| | Df(2L)ED758 | 33C1 - 33E4 | 367471 | 0.45 ( 0.13 ) | 0.23 ( 0.03 ) | 0.49 ( 0.23 ) | 0.36 ( 0.16 ) |
| | Df(2L)ED760 | 33B8 - 33E5 | 426429 | 0.44 ( 0.06 ) | 0.22 ( 0.12 ) | 0.49 ( 0.07 ) | 0.12 ( 0.06 ) |
| | Df(2L)ED761 | 33A2 - 33E5 | 627604 | 0.42 ( 0.28 ) | 0.10 ( 0.09 ) | 0.44 ( 0.27 ) | 0.14 ( 0.14 ) |
| | Df(2L)ED769 | 33E9 - 34A1 | 277041 | 0.48 ( 0.10 ) | 0.14 ( 0.06 ) | 0.42 ( 0.29 ) | 0.22 ( 0.13 ) |

Table S1. Continued.

| | | | | | | | | | | | |
|---|---|---|---|---|---|---|---|---|---|---|---|
| Df(2L)ED771 | 33E4 | - | 34A1 | 388303 | 0.49 (0.13) | | 0.35 (0.19) | | 0.20 (0.03) | | 0.25 (0.11) |
| Df(2L)ED773 | 33E9 | - | 34A3 | 429228 | 0.60 (0.30) | | 0.27 (0.18) | | 0.56 (0.16) | | 0.27 (0.17) |
| Df(2L)ED774 | 34A3 | - | 34A3 | 683 | 0.38 (0.10) | | 0.34 (0.15) | | 0.30 (0.04) | | 0.32 (0.10) |
| Df(2L)ED775 | 33B8 | - | 34A3 | 965018 | 0.50 (0.18) | | 0.19 (0.12) | | 0.62 (0.21) | | 0.10 (0.13) |
| Df(2L)ED776 | 33E4 | - | 34A3 | 540490 | 0.42 (0.14) | | 0.43 (0.16) | | 0.40 (0.18) | | 0.41 (0.13) |
| Df(2L)ED777 | 33E7 | - | 34A3 | 490576 | 0.50 (0.24) | | 0.08 (0.09) | | 0.46 (0.32) | | 0.06 (0.07) |
| Df(2L)ED778 | 33E9 | - | 34A7 | 619745 | 0.53 (0.13) | | 0.34 (0.20) | | 0.42 (0.14) | | 0.26 (0.09) |
| Df(2L)ED779 | 34A3 | - | 34A7 | 191200 | 0.55 (0.15) | | 0.28 (0.14) | | 0.56 (0.16) | | 0.29 (0.11) |
| Df(2L)ED780 | 33E4 | - | 34A7 | 731007 | 0.58 (0.15) | | 0.13 (0.11) | | 0.50 (0.12) | | 0.12 (0.05) |
| Df(2L)ED784 | 34A4 | - | 34B6 | 327612 | 0.32 (0.27) | | 0.10 (0.08) | | 0.26 (0.23) | | 0.16 (0.16) |
| Df(2L)ED791 | 34E1 | - | 35B4 | 811156 | 0.41 (0.10) | | 0.19 (0.03) | | 0.42 (0.07) | | 0.12 (0.07) |
| Df(2L)ED793 | 34E4 | - | 35B4 | 754489 | 0.39 (0.05) | | 0.35 (0.11) | | 0.52 (0.26) | | 0.30 (0.13) |
| Df(2L)ED796 | 35C1 | - | 35C4 | 152111 | 0.50 (0.15) | | 0.26 (0.15) | | 0.50 (0.10) | | 0.30 (0.18) |
| Df(2L)ED929 | 21B3 | - | 21B3 | 18484 | 0.52 (0.22) | | 0.27 (0.11) | | 0.50 (0.13) | | 0.37 (0.26) |
| Df(2L)ED1000 | 35B8 | - | 35D1 | 336213 | 0.61 (0.25) | | 0.45 (0.17) | | 0.55 (0.24) | | 0.37 (0.12) |
| Df(2L)ED1004 | 35B10 | - | 35D1 | 272692 | 0.58 (0.10) | | 0.26 (0.08) | | 0.43 (0.16) | | 0.26 (0.15) |
| Df(2L)ED1050 | 35B8 | - | 35D4 | 765231 | 0.40 (0.13) | | 0.07 (0.10) | | 0.26 (0.15) | | 0.06 (0.06) |
| Df(2L)ED1054 | 35B10 | - | 35D4 | 701710 | 0.61 (0.16) | | 0.35 (0.17) | | 0.51 (0.10) | | 0.43 (0.11) |
| Df(2L)ED1056 | 35D2 | - | 35D4 | 284125 | 0.44 (0.29) | | 0.37 (0.20) | | 0.51 (0.30) | | 0.26 (0.11) |
| Df(2L)ED1092 | 35F12 | - | 36A10 | 329835 | 0.34 (0.16) | | 0.11 (0.04) | | 0.46 (0.08) | | 0.13 (0.05) |
| Df(2L)ED1102 | 35F12 | - | 36A10 | 334647 | 0.48 (0.12) | | 0.27 (0.26) | | 0.46 (0.15) | | 0.30 (0.20) |
| Df(2L)ED1109 | 36A3 | - | 36A10 | 164790 | 0.82 (0.12) | | 0.42 (0.16) | | 0.63 (0.16) | | 0.55 (0.11) |
| Df(2L)ED1143 | 36A10 | - | 36B1 | 106247 | 0.22 (0.13) | | 0.13 (0.05) | | 0.23 (0.15) | | 0.10 (0.05) |
| Df(2L)ED1153 | 35F12 | - | 36B2 | 502171 | 0.50 (0.19) | | 0.08 (0.06) | | 0.45 (0.17) | | 0.06 (0.05) |
| Df(2L)ED1158 | 36B1 | - | 36C9 | 658852 | 0.54 (0.19) | | 0.13 (0.09) | | 0.60 (0.18) | | 0.26 (0.13) |
| Df(2L)ED1161 | 36A10 | - | 36C9 | 788014 | 0.31 (0.10) | | 0.24 (0.07) | | 0.34 (0.12) | | 0.10 (0.07) |
| Df(2L)ED1164 | 36A10 | - | 36C9 | 787566 | 0.46 (0.23) | | 0.27 (0.19) | | 0.52 (0.19) | | 0.18 (0.05) |
| Df(2L)ED1165 | 36C1 | - | 36C9 | 360447 | 0.45 (0.32) | | 0.30 (0.11) | | 0.47 (0.24) | | 0.26 (0.09) |
| Df(2L)ED1175 | 36C1 | - | 36C10 | 383436 | 0.47 (0.16) | | 0.35 (0.11) | | 0.36 (0.08) | | 0.26 (0.09) |
| Df(2L)ED1183 | 36E6 | - | 36F7 | 465553 | 0.30 (0.06) | | 0.03 (0.03) | | 0.34 (0.07) | | 0.07 (0.07) |
| Df(2L)ED1186 | 36E6 | - | 37A2 | 581220 | 0.34 (0.22) | | 0.14 (0.07) | | 0.34 (0.11) | | 0.12 (0.12) |
| Df(2L)ED1187 | 36F7 | - | 37A2 | 115693 | 0.62 (0.04) | | 0.44 (0.10) | | 0.00 (0.00) | | 0.00 (0.00) |
| Df(2L)ED1196 | 36E6 | - | 37B1 | 671892 | 0.65 (0.15) | | 0.20 (0.12) | | 0.49 (0.09) | | 0.22 (0.14) |
| Df(2L)ED1198 | 36F7 | - | 37B1 | 206365 | 0.57 (0.16) | | 0.38 (0.06) | | 0.54 (0.06) | | 0.25 (0.08) |
| Df(2L)ED1200 | 44D8 | - | 45B4 | 155049 | 0.37 (0.13) | | 0.27 (0.16) | | 0.36 (0.12) | | 0.19 (0.09) |
| Df(2L)ED1202 | 37B1 | - | 37C5 | 334948 | 0.37 (0.21) | | 0.09 (0.09) | | 0.29 (0.17) | | 0.06 (0.07) |
| Df(2L)ED1226 | 37B9 | - | 37E3 | 460658 | 0.35 (0.15) | | 0.21 (0.11) | | 0.26 (0.10) | | 0.20 (0.07) |
| Df(2L)ED1231 | 37C5 | - | 37E3 | 305616 | 0.34 (0.16) | | 0.12 (0.07) | | 0.18 (0.13) | | 0.15 (0.05) |
| Df(2L)ED1236 | 37B9 | - | 37E4 | 483659 | 0.53 (0.14) | | 0.17 (0.09) | | 0.55 (0.16) | | 0.26 (0.13) |
| Df(2L)ED1238 | 37C1 | - | 37E4 | 357571 | 0.36 (0.18) | | 0.32 (0.14) | | 0.34 (0.18) | | 0.23 (0.15) |
| Df(2L)ED1242 | 37E5 | - | 37F1 | 15994 | 0.65 (0.03) | | 0.34 (0.17) | | 0.58 (0.13) | | 0.19 (0.08) |
| Df(2L)ED1243 | 37B9 | - | 37F1 | 524349 | 0.54 (0.10) | | 0.38 (0.13) | | 0.39 (0.10) | | 0.29 (0.09) |
| Df(2L)ED1245 | 37C1 | - | 37F1 | 398261 | 0.46 (0.13) | | 0.25 (0.07) | | 0.46 (0.18) | | 0.26 (0.05) |
| Df(2L)ED1250 | 37E5 | - | 37F1 | 24869 | 0.71 (0.09) | | 0.32 (0.13) | | 0.66 (0.13) | | 0.18 (0.17) |
| Df(2L)ED1251 | 37B9 | - | 37F1 | 533224 | 0.56 (0.22) | | 0.21 (0.09) | | 0.32 (0.07) | | 0.10 (0.07) |
| Df(2L)ED1272 | 37C5 | - | 38A2 | 594884 | 0.31 (0.19) | | 0.15 (0.04) | | 0.30 (0.11) | | 0.15 (0.10) |
| Df(2L)ED1303 | 37E5 | - | 38C6 | 864775 | 0.44 (0.17) | | 0.36 (0.29) | | 0.42 (0.17) | | 0.22 (0.09) |
| Df(2L)ED1305 | 38B4 | - | 38C6 | 296988 | 0.49 (0.16) | | 0.17 (0.10) | | 0.48 (0.13) | | 0.09 (0.03) |
| Df(2L)ED1315 | 38B4 | - | 38F5 | 832122 | 0.50 (0.18) | | 0.09 (0.08) | | 0.38 (0.12) | | 0.10 (0.04) |
| Df(2L)ED1317 | 38D1 | - | 38F5 | 278939 | 0.30 (0.15) | | 0.24 (0.12) | | 0.24 (0.11) | | 0.21 (0.15) |
| Df(2L)ED1375 | 38F5 | - | 39D2 | 457289 | 0.50 (0.31) | | 0.38 (0.19) | | 0.48 (0.31) | | 0.26 (0.06) |
| Df(2L)ED1378 | 38F1 | - | 39D2 | 574133 | 0.32 (0.21) | | 0.19 (0.08) | | 0.35 (0.11) | | 0.14 (0.06) |
| Df(2L)ED1382 | 39B4 | - | 39D2 | 159357 | 0.46 (0.22) | | 0.29 (0.09) | | 0.46 (0.15) | | 0.18 (0.08) |
| Df(2L)ED1384 | 38F5 | - | 39D2 | 474447 | 0.62 (0.25) | | 0.28 (0.07) | | 0.61 (0.09) | | 0.42 (0.15) |
| Df(2L)ED1451 | 38F5 | - | 39E2 | 666875 | 0.48 (0.10) | | 0.37 (0.13) | | 0.63 (0.09) | | 0.26 (0.05) |
| Df(2L)ED1454 | 39E3 | - | 39E6 | 28361 | 0.54 (0.13) | | 0.23 (0.05) | | 0.49 (0.10) | | 0.17 (0.10) |
| Df(2L)ED1455 | 39A1 | - | 39E6 | 605551 | 0.51 (0.15) | | 0.00 ( - ) | | 0.44 (0.18) | | 0.00 ( - ) |
| Df(2L)ED1462 | 39B4 | - | 39E6 | 406785 | 0.42 (0.13) | | 0.11 (0.07) | | 0.28 (0.14) | | 0.13 (0.09) |
| Df(2L)ED1466 | 39E3 | - | 40A5 | 199232 | 0.46 (0.12) | | 0.38 (0.10) | | 0.57 (0.10) | | 0.30 (0.10) |
| Df(2L)ED1473 | 39B4 | - | 40A5 | 577656 | 0.53 (0.07) | | 0.26 (0.17) | | 0.42 (0.16) | | 0.23 (0.23) |
| Df(2L)ED2809 | 21B1 | - | 21B1 | 5306 | 0.82 (0.23) | | 0.42 (0.13) | | 0.80 (0.23) | | 0.54 (0.26) |
| Df(2L)ED4330 | 23C4 | - | 23C5 | 56592 | 0.54 (0.22) | | 0.32 (0.05) | | 0.48 (0.24) | | 0.22 (0.18) |
| Df(2L)ED4559 | 23C4 | - | 23F6 | 479077 | 0.70 (0.15) | | 0.42 (0.05) | | 0.60 (0.16) | | 0.38 (0.05) |
| Df(2L)ED4651 | 23B8 | - | 23F6 | 604377 | 0.50 (0.21) | | 0.22 (0.10) | | 0.33 (0.09) | | 0.15 (0.05) |
| Df(2L)ED5878 | 21B1 | - | 21B3 | 93755 | 0.68 (0.15) | | 0.25 (0.10) | | 0.69 (0.17) | | 0.22 (0.06) |
| Df(2L)ED6569 | 27A1 | - | 27C4 | 212193 | 0.51 (0.18) | | 0.34 (0.11) | | 0.63 (0.25) | | 0.29 (0.07) |
| Df(2L)ED7007 | 27A1 | - | 27C7 | 254709 | 0.52 (0.21) | | 0.30 (0.16) | | 0.58 (0.14) | | 0.18 (0.06) |
| Df(2L)ED7666 | 22F4 | - | 22F4 | 1188 | 0.49 (0.17) | | 0.31 (0.18) | | 0.47 (0.19) | | 0.30 (0.21) |
| Df(2L)ED7733 | 21E2 | - | 21F1 | 266285 | 0.48 (0.17) | | 0.27 (0.10) | | 0.56 (0.18) | | 0.30 (0.20) |
| Df(2L)ED7762 | 22A6 | - | 22D3 | 539713 | 0.34 (0.20) | | 0.02 (0.02) | | 0.41 (0.27) | | 0.02 (0.02) |
| Df(2L)ED7853 | 25A3 | - | 25B10 | 299273 | 0.43 (0.20) | | 0.42 (0.07) | | 0.55 (0.12) | | 0.43 (0.15) |
| Df(2L)ED8142 | 31E1 | - | 32A4 | 271218 | 0.64 (0.18) | | 0.38 (0.19) | | 0.56 (0.16) | | 0.33 (0.24) |
| Df(2L)ED8185 | 34E1 | - | 35A4 | 456009 | 0.66 (0.16) | | 0.42 (0.09) | | 0.73 (0.24) | | 0.43 (0.10) |
| Df(2L)ED8186 | 34E4 | - | 35A4 | 399342 | 0.70 (0.10) | | 0.36 (0.09) | | 0.60 (0.07) | | 0.24 (0.10) |
| Df(2L)ED8368 | 36B1 | - | 36B2 | 33618 | 0.30 (0.15) | | 0.22 (0.07) | | 0.36 (0.14) | | 0.18 (0.08) |
| Df(2L)ED8386 | 36C1 | - | 36C9 | 337487 | 0.59 (0.08) | | 0.29 (0.12) | | 0.71 (0.30) | | 0.18 (0.10) |
| Df(2L)ED12487 | 25C3 | - | 25F2 | 604135 | 0.51 (0.22) | | 0.10 (0.08) | | 0.52 (0.18) | | 0.05 (0.05) |
| Df(2L)ED12527 | 28C4 | - | 28D3 | 203671 | 0.54 (0.24) | | 0.28 (0.13) | | 0.58 (0.21) | | 0.26 (0.21) |
| Df(2L)ED13216 | 36A12 | - | 36B1 | 70720 | 0.32 (0.15) | | 0.22 (0.14) | | 0.30 (0.25) | | 0.16 (0.07) |

Table S1. Continued.

| | | | | | | | |
|---|---|---|---|---|---|---|---|
| 2R | Df(2R)ED1 | 53E9 - 53F8 | 70595 | 0.51 ( 0.20 ) | 0.34 ( 0.19 ) | 0.44 ( 0.21 ) | 0.40 ( 0.12 ) |
| | Df(2R)ED1482 | 42A8 - 42A11 | 96487 | 0.53 ( 0.18 ) | 0.26 ( 0.12 ) | 0.50 ( 0.24 ) | 0.18 ( 0.05 ) |
| | Df(2R)ED1484 | 42A2 - 42A14 | 351791 | 0.28 ( 0.18 ) | 0.22 ( 0.09 ) | 0.32 ( 0.22 ) | 0.11 ( 0.07 ) |
| | Df(2R)ED1552 | 42A11 - 42C7 | 608682 | 0.29 ( 0.15 ) | 0.09 ( 0.09 ) | 0.27 ( 0.10 ) | 0.14 ( 0.13 ) |
| | Df(2R)ED1612 | 42A13 - 42E6 | 829140 | 0.68 ( 0.23 ) | 0.09 ( 0.06 ) | 0.58 ( 0.15 ) | 0.07 ( 0.04 ) |
| | Df(2R)ED1618 | 42C3 - 43A1 | 518138 | 0.67 ( 0.26 ) | 0.37 ( 0.30 ) | 0.54 ( 0.19 ) | 0.28 ( 0.15 ) |
| | Df(2R)ED1673 | 42E4 - 43D3 | 547751 | 0.41 ( 0.07 ) | 0.34 ( 0.31 ) | 0.42 ( 0.16 ) | 0.22 ( 0.18 ) |
| | Df(2R)ED1715 | 43A4 - 43F1 | 589972 | 0.53 ( 0.19 ) | 0.42 ( 0.10 ) | 0.49 ( 0.11 ) | 0.20 ( 0.08 ) |
| | Df(2R)ED1725 | 43E4 - 44B5 | 542121 | 0.30 ( 0.08 ) | 0.16 ( 0.07 ) | 0.35 ( 0.12 ) | 0.16 ( 0.15 ) |
| | Df(2R)ED1735 | 43F8 - 44D4 | 638302 | 0.59 ( 0.22 ) | 0.31 ( 0.19 ) | 0.46 ( 0.08 ) | 0.23 ( 0.23 ) |
| | Df(2R)ED1742 | 44B8 - 44E3 | 549961 | 0.34 ( 0.07 ) | 0.24 ( 0.23 ) | 0.37 ( 0.13 ) | 0.14 ( 0.10 ) |
| | Df(2R)ED1770 | 14A8 - 14C6 | 551912 | 0.39 ( 0.25 ) | 0.22 ( 0.04 ) | 0.41 ( 0.23 ) | 0.17 ( 0.09 ) |
| | Df(2R)ED1791 | 44F7 - 45F1 | 630522 | 0.49 ( 0.11 ) | 0.33 ( 0.15 ) | 0.52 ( 0.15 ) | 0.46 ( 0.24 ) |
| | Df(2R)ED2076 | 47A10 - 47C1 | 343202 | 0.34 ( 0.27 ) | 0.40 ( 0.16 ) | 0.44 ( 0.14 ) | 0.33 ( 0.17 ) |
| | Df(2R)ED2098 | 47A7 - 47C6 | 482345 | 0.45 ( 0.19 ) | 0.22 ( 0.10 ) | 0.37 ( 0.12 ) | 0.13 ( 0.09 ) |
| | Df(2R)ED2155 | 47C6 - 47F8 | 503346 | 0.41 ( 0.12 ) | 0.28 ( 0.06 ) | 0.52 ( 0.16 ) | 0.29 ( 0.06 ) |
| | Df(2R)ED2219 | 47D6 - 48B6 | 467979 | 0.07 ( 0.08 ) | 0.03 ( 0.04 ) | 0.05 ( 0.05 ) | 0.05 ( 0.07 ) |
| | Df(2R)ED2222 | 47F13 - 48B6 | 212411 | 0.44 ( 0.23 ) | 0.10 ( 0.07 ) | 0.38 ( 0.18 ) | 0.11 ( 0.07 ) |
| | Df(2R)ED2247 | 53C9 - 53F10 | 388614 | 0.00 ( 0.00 ) | 0.01 ( 0.02 ) | 0.06 ( 0.13 ) | 0.00 ( 0.00 ) |
| | Df(2R)ED2308 | 49D3 - 49E7 | 216614 | 0.39 ( 0.13 ) | 0.32 ( 0.26 ) | 0.37 ( 0.11 ) | 0.25 ( 0.23 ) |
| | Df(2R)ED2311 | 49E4 - 49F10 | 277450 | 0.42 ( 0.24 ) | 0.08 ( 0.07 ) | 0.42 ( 0.21 ) | 0.08 ( 0.03 ) |
| | Df(2R)ED2354 | 50E6 - 51B1 | 315512 | 0.57 ( 0.26 ) | 0.18 ( 0.15 ) | 0.52 ( 0.06 ) | 0.20 ( 0.15 ) |
| | Df(2R)ED2423 | 51C5 - 51F11 | 520185 | 0.46 ( 0.22 ) | 0.32 ( 0.14 ) | 0.46 ( 0.24 ) | 0.27 ( 0.17 ) |
| | Df(2R)ED2426 | 51E2 - 52B1 | 482016 | 0.37 ( 0.21 ) | 0.22 ( 0.13 ) | 0.38 ( 0.22 ) | 0.22 ( 0.12 ) |
| | Df(2R)ED2436 | 51F11 - 52D11 | 627239 | 0.33 ( 0.21 ) | 0.37 ( 0.13 ) | 0.36 ( 0.26 ) | 0.27 ( 0.10 ) |
| | Df(2R)ED2457 | 52D11 - 52E7 | 129848 | 0.37 ( 0.17 ) | 0.12 ( 0.12 ) | 0.38 ( 0.14 ) | 0.02 ( 0.02 ) |
| | Df(2R)ED2487 | 52E6 - 53C4 | 261478 | 0.40 ( 0.04 ) | 0.13 ( 0.07 ) | 0.40 ( 0.17 ) | 0.18 ( 0.08 ) |
| | Df(2R)ED2748 | 53D11 - 53F8 | 268682 | 0.34 ( 0.11 ) | 0.26 ( 0.18 ) | 0.28 ( 0.21 ) | 0.15 ( 0.10 ) |
| | Df(2R)ED2751 | 53D14 - 53F8 | 240132 | 0.67 ( 0.31 ) | 0.25 ( 0.18 ) | 0.40 ( 0.16 ) | 0.12 ( 0.04 ) |
| | Df(2R)ED3181 | 57F10 - 57F10 | 524868 | 0.34 ( 0.11 ) | 0.24 ( 0.08 ) | 0.43 ( 0.14 ) | 0.36 ( 0.15 ) |
| | Df(2R)ED3610 | 54F1 - 55C8 | 561128 | 0.15 ( 0.09 ) | 0.01 ( 0.02 ) | 0.13 ( 0.05 ) | 0.02 ( 0.02 ) |
| | Df(2R)ED3636 | 55B8 - 55E3 | 498523 | 0.03 ( 0.05 ) | 0.04 ( 0.04 ) | 0.04 ( 0.04 ) | 0.03 ( 0.05 ) |
| | Df(2R)ED3683 | 55C2 - 56C4 | 940122 | 0.25 ( 0.16 ) | 0.19 ( 0.09 ) | 0.33 ( 0.22 ) | 0.10 ( 0.07 ) |
| | Df(2R)ED3728 | 56D10 - 56E2 | 264297 | 0.26 ( 0.11 ) | 0.08 ( 0.08 ) | 0.27 ( 0.10 ) | 0.10 ( 0.06 ) |
| | Df(2R)ED3791 | 57B1 - 57D4 | 552570 | 0.36 ( 0.21 ) | 0.30 ( 0.21 ) | 0.68 ( 0.16 ) | 0.30 ( 0.14 ) |
| | Df(2R)ED3921 | 57F9 - 57F10 | 11246 | 0.78 ( 0.13 ) | 0.28 ( 0.13 ) | 0.50 ( 0.13 ) | 0.26 ( 0.22 ) |
| | Df(2R)ED3923 | 57F6 - 57F10 | 67570 | 0.65 ( 0.19 ) | 0.45 ( 0.30 ) | 0.64 ( 0.09 ) | 0.31 ( 0.16 ) |
| | Df(2R)ED3943 | 37B9 - 37C5 | 688723 | 0.38 ( 0.09 ) | 0.08 ( 0.03 ) | 0.36 ( 0.16 ) | 0.08 ( 0.07 ) |
| | Df(2R)ED3952 | 58B10 - 58E5 | 386674 | 0.49 ( 0.23 ) | 0.20 ( 0.15 ) | 0.46 ( 0.12 ) | 0.27 ( 0.13 ) |
| | Df(2R)ED4061 | 60C8 - 60D13 | 270614 | 0.58 ( 0.26 ) | 0.27 ( 0.21 ) | 0.41 ( 0.13 ) | 0.18 ( 0.12 ) |
| | Df(2R)ED4071 | 60C8 - 60E8 | 540173 | 0.54 ( 0.24 ) | 0.03 ( 0.03 ) | 0.65 ( 0.33 ) | 0.03 ( 0.03 ) |
| | Df(2R)ED9039 | 48C5 - 48E4 | 283867 | 0.33 ( 0.16 ) | 0.31 ( 0.15 ) | 0.35 ( 0.14 ) | 0.20 ( 0.20 ) |
| | Df(2R)ED9045 | 48F5 - 49A7 | 212898 | 0.16 ( 0.17 ) | 0.14 ( 0.23 ) | 0.10 ( 0.13 ) | 0.03 ( 0.03 ) |
| 3L | Df(3L)ED201 | 91A5 - 91F1 | 224017 | 0.26 ( 0.11 ) | 0.06 ( 0.06 ) | 0.34 ( 0.21 ) | 0.02 ( 0.04 ) |
| | Df(3L)ED202 | 61C9 - 61F7 | 597642 | 0.60 ( 0.19 ) | 0.09 ( 0.08 ) | 0.52 ( 0.19 ) | 0.12 ( 0.08 ) |
| | Df(3L)ED207 | 61C9 - 62A6 | 829369 | 0.38 ( 0.27 ) | 0.23 ( 0.17 ) | 0.58 ( 0.23 ) | 0.23 ( 0.19 ) |
| | Df(3L)ED208 | 63C1 - 63F5 | 644000 | 0.23 ( 0.07 ) | 0.13 ( 0.09 ) | 0.18 ( 0.12 ) | 0.18 ( 0.12 ) |
| | Df(3L)ED210 | 64B9 - 64C13 | 804208 | 0.42 ( 0.12 ) | 0.26 ( 0.08 ) | 0.38 ( 0.15 ) | 0.14 ( 0.08 ) |
| | Df(3L)ED211 | 65A9 - 65B4 | 334624 | 0.30 ( 0.24 ) | 0.09 ( 0.05 ) | 0.31 ( 0.14 ) | 0.10 ( 0.08 ) |
| | Df(3L)ED215 | 69B5 - 69C4 | 86745 | 0.40 ( 0.11 ) | 0.22 ( 0.09 ) | 0.34 ( 0.08 ) | 0.30 ( 0.05 ) |
| | Df(3L)ED217 | 70F4 - 71E1 | 831026 | 0.30 ( 0.20 ) | 0.08 ( 0.07 ) | 0.36 ( 0.23 ) | 0.03 ( 0.04 ) |
| | Df(3L)ED218 | 71B1 - 71E1 | 575028 | 0.50 ( 0.25 ) | 0.18 ( 0.13 ) | 0.38 ( 0.15 ) | 0.12 ( 0.03 ) |
| | Df(3L)ED220 | 72D4 - 72F1 | 324193 | 0.63 ( 0.19 ) | 0.35 ( 0.16 ) | 0.70 ( 0.18 ) | 0.38 ( 0.13 ) |
| | Df(3L)ED223 | 73A1 - 73D5 | 439052 | 0.17 ( 0.07 ) | 0.03 ( 0.02 ) | 0.14 ( 0.15 ) | 0.05 ( 0.03 ) |
| | Df(3L)ED224 | 75B1 - 75C6 | 429316 | 0.30 ( 0.20 ) | 0.06 ( 0.05 ) | 0.34 ( 0.20 ) | 0.09 ( 0.08 ) |
| | Df(3L)ED225 | 75C1 - 75D4 | 435192 | 0.30 ( 0.08 ) | 0.26 ( 0.13 ) | 0.26 ( 0.13 ) | 0.20 ( 0.12 ) |
| | Df(3L)ED228 | 76A1 - 76D2 | 701102 | 0.19 ( 0.02 ) | 0.21 ( 0.09 ) | 0.26 ( 0.17 ) | 0.16 ( 0.05 ) |
| | Df(3L)ED230 | 79C2 - 80A4 | 699720 | 0.54 ( 0.18 ) | 0.04 ( 0.03 ) | 0.49 ( 0.15 ) | 0.06 ( 0.06 ) |
| | Df(3L)ED231 | 80B1 - 80C1 | 73704 | 0.48 ( 0.08 ) | 0.39 ( 0.11 ) | 0.56 ( 0.19 ) | 0.38 ( 0.19 ) |
| | Df(3L)ED4079 | 61A5 - 61B1 | 91461 | 0.37 ( 0.07 ) | 0.26 ( 0.07 ) | 0.42 ( 0.15 ) | 0.21 ( 0.08 ) |
| | Df(3L)ED4177 | 61C1 - 61E2 | 715336 | 0.34 ( 0.21 ) | 0.08 ( 0.05 ) | 0.29 ( 0.23 ) | 0.06 ( 0.08 ) |
| | Df(3L)ED4191 | 61C3 - 62A2 | 934664 | 0.22 ( 0.14 ) | 0.08 ( 0.06 ) | 0.28 ( 0.05 ) | 0.18 ( 0.09 ) |
| | Df(3L)ED4196 | 61C7 - 62A2 | 839354 | 0.37 ( 0.13 ) | 0.07 ( 0.05 ) | 0.34 ( 0.21 ) | 0.13 ( 0.12 ) |
| | Df(3L)ED4238 | 61C9 - 62A4 | 808192 | 0.46 ( 0.26 ) | 0.38 ( 0.10 ) | 0.40 ( 0.19 ) | 0.42 ( 0.11 ) |
| | Df(3L)ED4256 | 62A3 - 62A6 | 40559 | 0.43 ( 0.13 ) | 0.14 ( 0.05 ) | 0.44 ( 0.17 ) | 0.16 ( 0.11 ) |
| | Df(3L)ED4284 | 62B4 - 62B12 | 168110 | 0.50 ( 0.14 ) | 0.30 ( 0.11 ) | 0.53 ( 0.18 ) | 0.19 ( 0.06 ) |
| | Df(3L)ED4287 | 62B4 - 62E5 | 756319 | 0.36 ( 0.18 ) | 0.12 ( 0.05 ) | 0.38 ( 0.19 ) | 0.24 ( 0.05 ) |
| | Df(3L)ED4288 | 63A6 - 63B7 | 78264 | 0.38 ( 0.24 ) | 0.18 ( 0.06 ) | 0.36 ( 0.14 ) | 0.27 ( 0.12 ) |
| | Df(3L)ED4293 | 63C1 - 63C1 | 24226 | 0.36 ( 0.17 ) | 0.32 ( 0.09 ) | 0.38 ( 0.19 ) | 0.30 ( 0.09 ) |
| | Df(3L)ED4341 | 63F6 - 64B9 | 637145 | 0.33 ( 0.16 ) | 0.19 ( 0.12 ) | 0.19 ( 0.07 ) | 0.15 ( 0.12 ) |
| | Df(3L)ED4342 | 64A12 - 64B12 | 347385 | 0.42 ( 0.16 ) | 0.19 ( 0.13 ) | 0.46 ( 0.12 ) | 0.22 ( 0.11 ) |
| | Df(3L)ED4408 | 66A22 - 66C5 | 320467 | 0.18 ( 0.07 ) | 0.06 ( 0.02 ) | 0.27 ( 0.10 ) | 0.03 ( 0.02 ) |
| | Df(3L)ED4414 | 66D12 - 66E6 | 233661 | 0.39 ( 0.17 ) | 0.22 ( 0.13 ) | 0.35 ( 0.16 ) | 0.28 ( 0.13 ) |
| | Df(3L)ED4415 | 66D12 - 66E6 | 213016 | 0.22 ( 0.13 ) | 0.14 ( 0.10 ) | 0.26 ( 0.08 ) | 0.10 ( 0.13 ) |
| | Df(3L)ED4416 | 66E1 - 67B1 | 522145 | 0.42 ( 0.20 ) | 0.19 ( 0.08 ) | 0.48 ( 0.17 ) | 0.16 ( 0.11 ) |
| | Df(3L)ED4421 | 66D12 - 67B3 | 638749 | 0.34 ( 0.25 ) | 0.06 ( 0.02 ) | 0.22 ( 0.15 ) | 0.05 ( 0.05 ) |
| | Df(3L)ED4457 | 67E2 - 68A7 | 761858 | 0.38 ( 0.14 ) | 0.16 ( 0.08 ) | 0.17 ( 0.12 ) | 0.08 ( 0.07 ) |
| | Df(3L)ED4470 | 68A6 - 68E1 | 736241 | 0.53 ( 0.04 ) | 0.43 ( 0.17 ) | 0.61 ( 0.20 ) | 0.40 ( 0.19 ) |

Table S1. Continued.

| | | | | | | | |
|---|---|---|---|---|---|---|---|
| | Df(3L)ED4483 | 69A5 - 69D3 | 415994 | 0.42 ( 0.19 ) | 0.15 ( 0.07 ) | 0.34 ( 0.16 ) | 0.17 ( 0.07 ) |
| | Df(3L)ED4486 | 69C4 - 69F6 | 518066 | 0.25 ( 0.16 ) | 0.26 ( 0.15 ) | 0.26 ( 0.09 ) | 0.37 ( 0.09 ) |
| | Df(3L)ED4502 | 70A3 - 70C10 | 765786 | 0.35 ( 0.17 ) | 0.14 ( 0.10 ) | 0.51 ( 0.22 ) | 0.17 ( 0.10 ) |
| | Df(3L)ED4515 | 70C6 - 70C15 | 97860 | 0.52 ( 0.19 ) | 0.23 ( 0.06 ) | 0.34 ( 0.24 ) | 0.19 ( 0.12 ) |
| | Df(3L)ED4528 | 70C15 - 70D2 | 39982 | 0.56 ( 0.15 ) | 0.27 ( 0.08 ) | 0.55 ( 0.08 ) | 0.27 ( 0.12 ) |
| | Df(3L)ED4534 | 70C15 - 70D3 | 156653 | 0.30 ( 0.19 ) | 0.17 ( 0.12 ) | 0.36 ( 0.16 ) | 0.13 ( 0.09 ) |
| | Df(3L)ED4536 | 70C11 - 70D3 | 202563 | 0.38 ( 0.07 ) | 0.19 ( 0.03 ) | 0.35 ( 0.11 ) | 0.12 ( 0.11 ) |
| | Df(3L)ED4543 | 70C6 - 70F4 | 822815 | 0.16 ( 0.07 ) | 0.14 ( 0.13 ) | 0.33 ( 0.14 ) | 0.07 ( 0.02 ) |
| | Df(3L)ED4606 | 72D4 - 73C4 | 692639 | 0.19 ( 0.07 ) | 0.09 ( 0.02 ) | 0.34 ( 0.26 ) | 0.06 ( 0.07 ) |
| | Df(3L)ED4674 | 73B5 - 73E5 | 388134 | 0.42 ( 0.16 ) | 0.15 ( 0.03 ) | 0.25 ( 0.12 ) | 0.10 ( 0.08 ) |
| | Df(3L)ED4685 | 73D5 - 74E2 | 721094 | 0.22 ( 0.18 ) | 0.19 ( 0.13 ) | 0.21 ( 0.18 ) | 0.22 ( 0.05 ) |
| | Df(3L)ED4710 | 74D1 - 75B11 | 651836 | 0.29 ( 0.12 ) | 0.07 ( 0.06 ) | 0.30 ( 0.14 ) | 0.09 ( 0.08 ) |
| | Df(3L)ED4743 | 75D4 - 75D8 | 133616 | 0.54 ( 0.13 ) | 0.42 ( 0.17 ) | 0.70 ( 0.14 ) | 0.44 ( 0.18 ) |
| | Df(3L)ED4744 | 75D8 - 75E1 | 14368 | 0.38 ( 0.06 ) | 0.37 ( 0.12 ) | 0.38 ( 0.12 ) | 0.26 ( 0.18 ) |
| | Df(3L)ED4782 | 75F2 - 76A1 | 174808 | 0.62 ( 0.09 ) | 0.52 ( 0.22 ) | 0.42 ( 0.10 ) | 0.39 ( 0.13 ) |
| | Df(3L)ED4786 | 75F7 - 76A5 | 194711 | 0.42 ( 0.22 ) | 0.34 ( 0.16 ) | 0.40 ( 0.07 ) | 0.28 ( 0.19 ) |
| | Df(3L)ED4789 | 76A1 - 76A5 | 124956 | 0.68 ( 0.17 ) | 0.54 ( 0.17 ) | 0.64 ( 0.21 ) | 0.44 ( 0.16 ) |
| | Df(3L)ED4799 | 76A1 - 76B3 | 311466 | 0.53 ( 0.10 ) | 0.31 ( 0.12 ) | 0.57 ( 0.09 ) | 0.30 ( 0.09 ) |
| | Df(3L)ED4858 | 76D3 - 77C1 | 506447 | 0.30 ( 0.12 ) | 0.13 ( 0.10 ) | 0.34 ( 0.21 ) | 0.09 ( 0.08 ) |
| | Df(3L)ED4957 | 78C3 - 78F1 | 530381 | 0.38 ( 0.22 ) | 0.10 ( 0.10 ) | 0.42 ( 0.28 ) | 0.15 ( 0.09 ) |
| | Df(3L)ED4978 | 78D5 - 79A2 | 346878 | 0.37 ( 0.20 ) | 0.24 ( 0.06 ) | 0.37 ( 0.14 ) | 0.31 ( 0.16 ) |
| | Df(3L)ED5013 | 80A1 - 80B1 | 150650 | 0.63 ( 0.10 ) | 0.25 ( 0.13 ) | 0.57 ( 0.08 ) | 0.25 ( 0.10 ) |
| | Df(3L)ED5017 | 80A4 - 80C2 | 162804 | 0.22 ( 0.14 ) | 0.02 ( 0.02 ) | 0.35 ( 0.11 ) | 0.10 ( 0.13 ) |
| 3R | Df(3R)ED2 | 21E2 - 21E2 | 697540 | 0.30 ( 0.02 ) | 0.21 ( 0.08 ) | 0.36 ( 0.19 ) | 0.24 ( 0.09 ) |
| | Df(3R)ED5020 | 82A3 - 82B1 | 108705 | 0.66 ( 0.09 ) | 0.40 ( 0.23 ) | 0.71 ( 0.13 ) | 0.28 ( 0.15 ) |
| | Df(3R)ED5021 | 82A1 - 82B1 | 193118 | 0.73 ( 0.26 ) | 0.09 ( 0.05 ) | 0.68 ( 0.24 ) | 0.06 ( 0.05 ) |
| | Df(3R)ED5046 | 82A1 - 82D3 | 541858 | 0.62 ( 0.20 ) | 0.26 ( 0.13 ) | 0.66 ( 0.27 ) | 0.16 ( 0.08 ) |
| | Df(3R)ED5066 | 82D1 - 82E4 | 302797 | 0.54 ( 0.16 ) | 0.34 ( 0.13 ) | 0.47 ( 0.20 ) | 0.34 ( 0.12 ) |
| | Df(3R)ED5071 | 82A1 - 82E4 | 755409 | 0.30 ( 0.20 ) | 0.29 ( 0.10 ) | 0.23 ( 0.12 ) | 0.23 ( 0.12 ) |
| | Df(3R)ED5092 | 82A3 - 82E8 | 805399 | 0.30 ( 0.13 ) | 0.11 ( 0.05 ) | 0.22 ( 0.14 ) | 0.05 ( 0.07 ) |
| | Df(3R)ED5095 | 82D1 - 82E8 | 437200 | 0.36 ( 0.12 ) | 0.25 ( 0.12 ) | 0.46 ( 0.15 ) | 0.28 ( 0.07 ) |
| | Df(3R)ED5100 | 82A1 - 82E8 | 889812 | 0.29 ( 0.05 ) | 0.18 ( 0.21 ) | 0.18 ( 0.06 ) | 0.12 ( 0.07 ) |
| | Df(3R)ED5138 | 82D5 - 82F8 | 483811 | 0.26 ( 0.19 ) | 0.14 ( 0.05 ) | 0.35 ( 0.15 ) | 0.14 ( 0.07 ) |
| | Df(3R)ED5142 | 82B3 - 82F8 | 811587 | 0.34 ( 0.14 ) | 0.28 ( 0.14 ) | 0.20 ( 0.13 ) | 0.26 ( 0.08 ) |
| | Df(3R)ED5147 | 82E8 - 83A1 | 280684 | 0.61 ( 0.10 ) | 0.08 ( 0.06 ) | 0.66 ( 0.18 ) | 0.12 ( 0.09 ) |
| | Df(3R)ED5156 | 82F8 - 83A4 | 193919 | 0.62 ( 0.17 ) | 0.42 ( 0.13 ) | 0.48 ( 0.16 ) | 0.26 ( 0.16 ) |
| | Df(3R)ED5177 | 83B4 - 83B6 | 23466 | 0.40 ( 0.32 ) | 0.12 ( 0.10 ) | 0.52 ( 0.16 ) | 0.23 ( 0.16 ) |
| | Df(3R)ED5187 | 83B7 - 83B8 | 6020 | 0.31 ( 0.18 ) | 0.06 ( 0.04 ) | 0.51 ( 0.12 ) | 0.11 ( 0.04 ) |
| | Df(3R)ED5196 | 83B9 - 83D2 | 323565 | 0.74 ( 0.15 ) | 0.06 ( 0.06 ) | 0.69 ( 0.26 ) | 0.10 ( 0.12 ) |
| | Df(3R)ED5197 | 83B7 - 83D2 | 359362 | 0.41 ( 0.18 ) | 0.10 ( 0.12 ) | 0.34 ( 0.07 ) | 0.02 ( 0.02 ) |
| | Df(3R)ED5220 | 84E6 - 84E11 | 116309 | 0.46 ( 0.16 ) | 0.26 ( 0.18 ) | 0.38 ( 0.13 ) | 0.31 ( 0.13 ) |
| | Df(3R)ED5221 | 84C4 - 84E11 | 965801 | 0.60 ( 0.12 ) | 0.26 ( 0.12 ) | 0.51 ( 0.12 ) | 0.20 ( 0.05 ) |
| | Df(3R)ED5223 | 84D9 - 84E11 | 602379 | 0.27 ( 0.16 ) | 0.30 ( 0.09 ) | 0.22 ( 0.10 ) | 0.27 ( 0.14 ) |
| | Df(3R)ED5230 | 84E6 - 85A5 | 675360 | 0.62 ( 0.28 ) | 0.67 ( 0.19 ) | 0.46 ( 0.19 ) | 0.50 ( 0.16 ) |
| | Df(3R)ED5296 | 84F6 - 85C3 | 806270 | 0.30 ( 0.13 ) | 0.17 ( 0.13 ) | 0.41 ( 0.21 ) | 0.14 ( 0.15 ) |
| | Df(3R)ED5301 | 85C3 - 85C3 | 22497 | 0.64 ( 0.07 ) | 0.40 ( 0.24 ) | 0.62 ( 0.14 ) | 0.39 ( 0.14 ) |
| | Df(3R)ED5327 | 85D1 - 85D1 | 2719 | 0.58 ( 0.12 ) | 0.18 ( 0.12 ) | 0.68 ( 0.14 ) | 0.24 ( 0.05 ) |
| | Df(3R)ED5330 | 85A5 - 85D1 | 560209 | 0.21 ( 0.13 ) | 0.07 ( 0.05 ) | 0.17 ( 0.16 ) | 0.04 ( 0.03 ) |
| | Df(3R)ED5331 | 85C3 - 85D1 | 195601 | 0.54 ( 0.27 ) | 0.40 ( 0.13 ) | 0.57 ( 0.08 ) | 0.35 ( 0.14 ) |
| | Df(3R)ED5339 | 85D1 - 85D11 | 125299 | 0.75 ( 0.12 ) | 0.13 ( 0.10 ) | 0.66 ( 0.10 ) | 0.24 ( 0.10 ) |
| | Df(3R)ED5416 | 85D16 - 85E6 | 335297 | 0.51 ( 0.24 ) | 0.21 ( 0.11 ) | 0.46 ( 0.15 ) | 0.22 ( 0.11 ) |
| | Df(3R)ED5428 | 85E1 - 85F8 | 417820 | 0.37 ( 0.08 ) | 0.30 ( 0.06 ) | 0.34 ( 0.17 ) | 0.27 ( 0.07 ) |
| | Df(3R)ED5438 | 85E5 - 85F8 | 321934 | 0.38 ( 0.17 ) | 0.31 ( 0.17 ) | 0.43 ( 0.16 ) | 0.43 ( 0.18 ) |
| | Df(3R)ED5472 | 85F16 - 86B1 | 180223 | 0.46 ( 0.22 ) | 0.45 ( 0.20 ) | 0.56 ( 0.13 ) | 0.42 ( 0.16 ) |
| | Df(3R)ED5474 | 85F11 - 86B1 | 241312 | 0.40 ( 0.21 ) | 0.41 ( 0.15 ) | 0.42 ( 0.07 ) | 0.40 ( 0.19 ) |
| | Df(3R)ED5495 | 85F16 - 86C7 | 716259 | 0.27 ( 0.15 ) | 0.07 ( 0.09 ) | 0.22 ( 0.10 ) | 0.13 ( 0.09 ) |
| | Df(3R)ED5506 | 86C7 - 86D5 | 287750 | 0.76 ( 0.06 ) | 0.34 ( 0.12 ) | 0.70 ( 0.12 ) | 0.19 ( 0.13 ) |
| | Df(3R)ED5511 | 86C7 - 86D9 | 359178 | 0.93 ( 0.30 ) | 0.87 ( 0.15 ) | 1.10 ( 0.27 ) | 0.71 ( 0.17 ) |
| | Df(3R)ED5514 | 86C7 - 86E11 | 684255 | 0.22 ( 0.07 ) | 0.15 ( 0.14 ) | 0.19 ( 0.04 ) | 0.18 ( 0.15 ) |
| | Df(3R)ED5516 | 86D8 - 86E13 | 385730 | 0.31 ( 0.07 ) | 0.42 ( 0.16 ) | 0.23 ( 0.10 ) | 0.35 ( 0.18 ) |
| | Df(3R)ED5518 | 86C7 - 86E13 | 734902 | 0.08 ( 0.05 ) | 0.01 ( 0.02 ) | 0.14 ( 0.06 ) | 0.00 ( 0.00 ) |
| | Df(3R)ED5519 | 86E11 - 86E13 | 53930 | 0.58 ( 0.11 ) | 0.46 ( 0.20 ) | 0.58 ( 0.11 ) | 0.32 ( 0.11 ) |
| | Df(3R)ED5554 | 87B5 - 87B11 | 162903 | 0.71 ( 0.22 ) | 0.38 ( 0.09 ) | 0.74 ( 0.29 ) | 0.34 ( 0.16 ) |
| | Df(3R)ED5558 | 86F9 - 87B11 | 615275 | 0.58 ( 0.17 ) | 0.42 ( 0.05 ) | 0.61 ( 0.19 ) | 0.43 ( 0.11 ) |
| | Df(3R)ED5559 | 86E11 - 87B11 | 874834 | 0.42 ( 0.20 ) | 0.03 ( 0.03 ) | 0.40 ( 0.17 ) | 0.16 ( 0.05 ) |
| | Df(3R)ED5573 | 87B5 - 87B13 | 196465 | 0.38 ( 0.23 ) | 0.23 ( 0.08 ) | 0.28 ( 0.12 ) | 0.14 ( 0.08 ) |
| | Df(3R)ED5577 | 86F9 - 87B13 | 648837 | 0.66 ( 0.10 ) | 0.02 ( 0.04 ) | 0.46 ( 0.12 ) | 0.03 ( 0.03 ) |
| | Df(3R)ED5591 | 87B7 - 87C7 | 369479 | 0.74 ( 0.22 ) | 0.34 ( 0.17 ) | 0.57 ( 0.16 ) | 0.34 ( 0.22 ) |
| | Df(3R)ED5608 | 87C7 - 87D7 | 275690 | 0.10 ( 0.02 ) | 0.02 ( 0.02 ) | 0.27 ( 0.10 ) | 0.02 ( 0.02 ) |
| | Df(3R)ED5610 | 87B11 - 87D7 | 551659 | 0.56 ( 0.06 ) | 0.07 ( 0.03 ) | 0.73 ( 0.21 ) | 0.13 ( 0.10 ) |
| | Df(3R)ED5612 | 87C7 - 87F6 | 925149 | 0.21 ( 0.15 ) | 0.10 ( 0.06 ) | 0.20 ( 0.11 ) | 0.10 ( 0.07 ) |
| | Df(3R)ED5613 | 87E3 - 87F6 | 385385 | 0.50 ( 0.17 ) | 0.18 ( 0.14 ) | 0.50 ( 0.11 ) | 0.12 ( 0.13 ) |
| | Df(3R)ED5622 | 87F10 - 88A4 | 300090 | 0.22 ( 0.12 ) | 0.12 ( 0.09 ) | 0.21 ( 0.11 ) | 0.12 ( 0.06 ) |
| | Df(3R)ED5623 | 87E3 - 88A4 | 724163 | 0.15 ( 0.08 ) | 0.02 ( 0.04 ) | 0.14 ( 0.08 ) | 0.01 ( 0.02 ) |
| | Df(3R)ED5634 | 88A4 - 88B1 | 260040 | 0.32 ( 0.11 ) | 0.22 ( 0.08 ) | 0.38 ( 0.13 ) | 0.12 ( 0.11 ) |
| | Df(3R)ED5642 | 87F10 - 88C2 | 797952 | 1.04 ( 0.21 ) | 0.68 ( 0.25 ) | 1.03 ( 0.20 ) | 0.71 ( 0.24 ) |
| | Df(3R)ED5644 | 88A4 - 88C9 | 607806 | 0.61 ( 0.20 ) | 0.34 ( 0.11 ) | 0.60 ( 0.16 ) | 0.34 ( 0.16 ) |
| | Df(3R)ED5657 | 88D1 - 88D7 | 221350 | 0.26 ( 0.10 ) | 0.24 ( 0.08 ) | 0.42 ( 0.06 ) | 0.31 ( 0.05 ) |
| | Df(3R)ED5660 | 88D1 - 88E1 | 396848 | 0.45 ( 0.20 ) | 0.23 ( 0.15 ) | 0.42 ( 0.15 ) | 0.33 ( 0.10 ) |

Table S1. Continued.

| | | | | | | | | | | | | |
|---|---|---|---|---|---|---|---|---|---|---|---|---|
| | Df(3R)ED5662 | 88D1 - | 88E2 | 434545 | 0.38 | (0.14) | 0.14 | (0.11) | 0.34 | (0.14) | 0.17 | (0.11) |
| | Df(3R)ED5664 | 88D1 - | 88E3 | 531540 | 0.53 | (0.14) | 0.02 | (0.04) | 0.32 | (0.11) | 0.02 | (0.04) |
| | Df(3R)ED5688 | 88E12 - | 88F1 | 37068 | 0.34 | (0.13) | 0.08 | (0.05) | 0.30 | (0.06) | 0.14 | (0.06) |
| | Df(3R)ED5705 | 88E12 - | 89A5 | 502138 | 0.14 | (0.08) | 0.08 | (0.06) | 0.10 | (0.07) | 0.02 | (0.04) |
| | Df(3R)ED5780 | 89E11 - | 90C1 | 625324 | 0.37 | (0.13) | 0.18 | (0.19) | 0.42 | (0.10) | 0.25 | (0.11) |
| | Df(3R)ED5781 | 89E13 - | 90C1 | 562695 | 0.57 | (0.20) | 0.31 | (0.13) | 0.53 | (0.23) | 0.27 | (0.07) |
| | Df(3R)ED5785 | 90C2 - | 90D1 | 225960 | 0.64 | (0.21) | 0.24 | (0.15) | 0.57 | (0.19) | 0.30 | (0.20) |
| | Df(3R)ED5807 | 90C2 - | 91A5 | 681121 | 0.31 | (0.18) | 0.07 | (0.06) | 0.15 | (0.05) | 0.05 | (0.04) |
| | Df(3R)ED5815 | 90F4 - | 91B8 | 491112 | 0.04 | (0.04) | 0.02 | (0.02) | 0.01 | (0.02) | 0.02 | (0.04) |
| | Df(3R)ED5911 | 91C5 - | 91F8 | 422856 | 0.50 | (0.14) | 0.30 | (0.15) | 0.46 | (0.19) | 0.14 | (0.07) |
| | Df(3R)ED5938 | 91D4 - | 92A11 | 735402 | 0.14 | (0.06) | 0.04 | (0.04) | 0.11 | (0.06) | 0.04 | (0.00) |
| | Df(3R)ED6025 | 92A11 - | 92E2 | 666791 | 0.53 | (0.17) | 0.00 | (0.00) | 0.38 | (0.05) | 0.01 | (0.02) |
| | Df(3R)ED6027 | 92B3 - | 92E2 | 472646 | 0.65 | (0.28) | 0.30 | (0.13) | 0.70 | (0.08) | 0.34 | (0.18) |
| | Df(3R)ED6052 | 93D4 - | 93D8 | 68869 | 0.37 | (0.07) | 0.22 | (0.13) | 0.42 | (0.22) | 0.18 | (0.09) |
| | Df(3R)ED6058 | 93D4 - | 93F6 | 423105 | 0.38 | (0.21) | 0.13 | (0.09) | 0.39 | (0.19) | 0.07 | (0.03) |
| | Df(3R)ED6076 | 93E10 - | 94A1 | 409323 | 0.56 | (0.27) | 0.26 | (0.10) | 0.30 | (0.14) | 0.22 | (0.08) |
| | Df(3R)ED6079 | 94A1 - | 94A2 | 91507 | 0.55 | (0.23) | 0.37 | (0.10) | 0.66 | (0.15) | 0.30 | (0.08) |
| | Df(3R)ED6085 | 93F14 - | 94B5 | 706744 | 0.29 | (0.18) | 0.18 | (0.14) | 0.22 | (0.16) | 0.18 | (0.07) |
| | Df(3R)ED6090 | 94A1 - | 94C1 | 656195 | 0.27 | (0.11) | 0.12 | (0.06) | 0.34 | (0.16) | 0.17 | (0.10) |
| | Df(3R)ED6091 | 94B5 - | 94C4 | 138626 | 0.53 | (0.13) | 0.11 | (0.04) | 0.56 | (0.09) | 0.17 | (0.08) |
| | Df(3R)ED6093 | 94A2 - | 94C4 | 592519 | 0.37 | (0.16) | 0.17 | (0.10) | 0.19 | (0.07) | 0.16 | (0.09) |
| | Df(3R)ED6096 | 94B5 - | 94E7 | 634288 | 0.14 | (0.11) | 0.02 | (0.02) | 0.06 | (0.05) | 0.02 | (0.02) |
| | Df(3R)ED6103 | 94D3 - | 94E9 | 359862 | 0.32 | (0.15) | 0.14 | (0.08) | 0.25 | (0.06) | 0.12 | (0.05) |
| | Df(3R)ED6105 | 94E9 - | 94E11 | 37549 | 0.26 | (0.05) | 0.12 | (0.10) | 0.38 | (0.17) | 0.10 | (0.08) |
| | Df(3R)ED6116 | 95B4 - | 95C1 | 46434 | 0.57 | (0.14) | 0.27 | (0.09) | 0.48 | (0.28) | 0.26 | (0.15) |
| | Df(3R)ED6119 | 95C8 - | 95C12 | 35707 | 0.28 | (0.14) | 0.14 | (0.04) | 0.27 | (0.15) | 0.12 | (0.08) |
| | Df(3R)ED6144 | 95C8 - | 95D1 | 55833 | 0.57 | (0.28) | 0.10 | (0.08) | 0.54 | (0.37) | 0.14 | (0.08) |
| | Df(3R)ED6150 | 95D1 - | 95D11 | 114310 | 0.42 | (0.24) | 0.27 | (0.26) | 0.50 | (0.16) | 0.17 | (0.12) |
| | Df(3R)ED6155 | 95B4 - | 95D11 | 283236 | 0.32 | (0.10) | 0.19 | (0.11) | 0.33 | (0.09) | 0.20 | (0.12) |
| | Df(3R)ED6168 | 95D1 - | 95F8 | 328141 | 0.24 | (0.13) | 0.10 | (0.07) | 0.21 | (0.06) | 0.04 | (0.04) |
| | Df(3R)ED6187 | 95D10 - | 96A7 | 492295 | 0.14 | (0.08) | 0.12 | (0.03) | 0.33 | (0.17) | 0.15 | (0.10) |
| | Df(3R)ED6220 | 96A7 - | 96C3 | 639975 | 0.74 | (0.23) | 0.50 | (0.07) | 0.62 | (0.17) | 0.39 | (0.12) |
| | Df(3R)ED6232 | 96F10 - | 97D2 | 762106 | 0.20 | (0.09) | 0.14 | (0.07) | 0.12 | (0.11) | 0.06 | (0.02) |
| | Df(3R)ED6235 | 97B9 - | 97D12 | 445273 | 0.54 | (0.18) | 0.48 | (0.13) | 0.51 | (0.07) | 0.60 | (0.19) |
| | Df(3R)ED6242 | 97E4 - | 97E11 | 123525 | 0.29 | (0.03) | 0.17 | (0.05) | 0.35 | (0.10) | 0.07 | (0.02) |
| | Df(3R)ED6255 | 97D2 - | 97F1 | 482865 | 0.30 | (0.08) | 0.14 | (0.06) | 0.33 | (0.09) | 0.14 | (0.02) |
| | Df(3R)ED6265 | 97E2 - | 98A7 | 467511 | 0.59 | (0.28) | 0.18 | (0.17) | 0.61 | (0.28) | 0.14 | (0.05) |
| | Df(3R)ED6277 | 98B6 - | 98B6 | 10924 | 0.65 | (0.20) | 0.15 | (0.07) | 0.46 | (0.09) | 0.14 | (0.09) |
| | Df(3R)ED6290 | 98C3 - | 98E5 | 485726 | 0.48 | (0.20) | 0.16 | (0.09) | 0.59 | (0.27) | 0.18 | (0.08) |
| | Df(3R)ED6310 | 98F12 - | 99B2 | 373258 | 0.51 | (0.08) | 0.25 | (0.20) | 0.65 | (0.31) | 0.13 | (0.18) |
| | Df(3R)ED6315 | 99B10 - | 99C1 | 17077 | 0.34 | (0.16) | 0.27 | (0.06) | 0.32 | (0.08) | 0.25 | (0.12) |
| | Df(3R)ED6316 | 99A5 - | 99C1 | 527344 | 0.25 | (0.14) | 0.11 | (0.04) | 0.36 | (0.14) | 0.13 | (0.04) |
| | Df(3R)ED6332 | 99E4 - | 99F2 | 111366 | 0.30 | (0.09) | 0.36 | (0.19) | 0.36 | (0.09) | 0.33 | (0.07) |
| | Df(3R)ED6346 | 100A5 - | 100B1 | 265322 | 0.18 | (0.11) | 0.19 | (0.15) | 0.26 | (0.10) | 0.23 | (0.13) |
| | Df(3R)ED6361 | 100C7 - | 100E3 | 469313 | 0.34 | (0.30) | 0.11 | (0.09) | 0.26 | (0.08) | 0.18 | (0.04) |
| | Df(3R)ED6362 | 100E1 - | 100E3 | 141893 | 0.47 | (0.12) | 0.19 | (0.10) | 0.39 | (0.15) | 0.22 | (0.10) |
| | Df(3R)ED7665 | 84B4 - | 84E11 | 1003556 | 0.07 | (0.05) | 0.16 | (0.14) | 0.08 | (0.04) | 0.09 | (0.06) |
| | Df(3R)ED10257 | 83A7 - | 83B4 | 81922 | 0.33 | (0.12) | 0.13 | (0.07) | 0.30 | (0.14) | 0.11 | (0.09) |
| | Df(3R)ED10549 | 88D6 - | 88D7 | 17163 | 0.59 | (0.09) | 0.30 | (0.09) | 0.44 | (0.10) | 0.34 | (0.20) |
| | Df(3R)ED10555 | 88C9 - | 88D8 | 361038 | 0.82 | (0.05) | 0.40 | (0.20) | 0.65 | (0.17) | 0.61 | (0.11) |
| | Df(3R)ED10556 | 88D6 - | 88E1 | 192661 | 0.53 | (0.18) | 0.27 | (0.17) | 0.35 | (0.12) | 0.21 | (0.14) |
| | Df(3R)ED10557 | 88D6 - | 88E2 | 230358 | 0.44 | (0.22) | 0.21 | (0.07) | 0.48 | (0.34) | 0.16 | (0.13) |
| | Df(3R)ED10561 | 88E2 - | 88E2 | 25799 | 0.42 | (0.17) | 0.21 | (0.04) | 0.42 | (0.08) | 0.14 | (0.07) |
| | Df(3R)ED10564 | 88D6 - | 88E3 | 327353 | 0.36 | (0.22) | 0.14 | (0.12) | 0.33 | (0.27) | 0.14 | (0.09) |
| | Df(3R)ED10566 | 88E2 - | 88E5 | 118112 | 0.64 | (0.16) | 0.23 | (0.17) | 0.66 | (0.11) | 0.16 | (0.07) |
| | Df(3R)ED10639 | 89B7 - | 89D2 | 268307 | 0.30 | (0.12) | 0.21 | (0.06) | 0.24 | (0.10) | 0.04 | (0.03) |
| | Df(3R)ED10642 | 89C7 - | 89D5 | 171514 | 0.37 | (0.16) | 0.27 | (0.07) | 0.38 | (0.13) | 0.22 | (0.10) |
| | Df(3R)ED10811 | 93A4 - | 93B8 | 111808 | 0.24 | (0.14) | 0.10 | (0.06) | 0.31 | (0.07) | 0.13 | (0.06) |
| | Df(3R)ED10820 | 93A4 - | 93B12 | 162720 | 0.39 | (0.08) | 0.26 | (0.10) | 0.52 | (0.03) | 0.32 | (0.08) |
| | Df(3R)ED10838 | 93C1 - | 93D4 | 162185 | 0.44 | (0.23) | 0.07 | (0.07) | 0.60 | (0.38) | 0.05 | (0.05) |
| | Df(3R)ED10893 | 95C8 - | 95E1 | 217754 | 0.47 | (0.12) | 0.29 | (0.03) | 0.34 | (0.09) | 0.19 | (0.17) |
| | Df(3R)ED10894 | 95A7 - | 95E1 | 435407 | 0.45 | (0.10) | 0.37 | (0.18) | 0.50 | (0.10) | 0.29 | (0.08) |
| | Df(3R)ED10946 | 96B20 - | 96D1 | 221386 | 0.40 | (0.11) | 0.25 | (0.12) | 0.37 | (0.13) | 0.27 | (0.10) |
| | Df(3R)ED10953 | 96C6 - | 96D1 | 70912 | 0.26 | (0.07) | 0.38 | (0.07) | 0.38 | (0.10) | 0.34 | (0.07) |
| | Df(3R)ED10961 | 97E11 - | 97F1 | 19770 | 0.46 | (0.16) | 0.28 | (0.11) | 0.46 | (0.06) | 0.22 | (0.09) |
| | Df(3R)ED10966 | 97E11 - | 97F1 | 28417 | 0.50 | (0.21) | 0.22 | (0.12) | 0.37 | (0.05) | 0.27 | (0.09) |
| | Df(3R)ED10970 | 97E11 - | 98B5 | 652492 | 0.18 | (0.12) | 0.14 | (0.07) | 0.22 | (0.07) | 0.06 | (0.06) |
| | Df(3R)ED10993 | 99B10 - | 99C2 | 41267 | 0.32 | (0.10) | 0.16 | (0.10) | 0.26 | (0.07) | 0.24 | (0.16) |
| | Df(3R)ED13102 | 99B1 - | 99B10 | 279997 | 0.37 | (0.07) | 0.18 | (0.05) | 0.24 | (0.12) | 0.16 | (0.08) |
| X | Df(1)ED404 | 1D2 - | 1E3 | 200503 | 0.30 | (0.07) | 0.26 | (0.16) | 0.00 | (0.00) | 0.00 | (0.00) |
| | Df(1)ED409 | 2C7 - | 2F5 | 275404 | 0.19 | (0.11) | 0.14 | (0.12) | 0.02 | (0.05) | 0.00 | (0.00) |
| | Df(1)ED411 | 3A3 - | 3A8 | 172827 | 0.27 | (0.13) | 0.19 | (0.11) | 0.02 | (0.05) | 0.00 | (0.00) |
| | Df(1)ED418 | 5C7 - | 5E4 | 377712 | 0.24 | (0.30) | 0.22 | (0.18) | 0.01 | (0.02) | 0.00 | (0.00) |
| | Df(1)ED429 | 9D3 - | 9D3 | 38567 | 0.84 | (0.27) | 0.38 | (0.12) | 0.78 | (0.25) | 0.38 | (0.18) |
| | Df(1)ED447 | 17C1 - | 17F1 | 356796 | 0.45 | (0.23) | 0.27 | (0.05) | 0.01 | (0.02) | 0.00 | (0.00) |
| | Df(1)ED6396 | 1B5 - | 1B8 | 30101 | 0.34 | (0.08) | 0.22 | (0.08) | 0.01 | (0.02) | 0.00 | (0.00) |
| | Df(1)ED6443 | 1B14 - | 1E1 | 370684 | 0.35 | (0.12) | 0.22 | (0.11) | 0.00 | (0.00) | 0.01 | (0.02) |
| | Df(1)ED6574 | 2E1 - | 3A2 | 203136 | 0.46 | (0.20) | 0.34 | (0.21) | 0.00 | (0.00) | 0.00 | (0.00) |
| | Df(1)ED6579 | 3A6 - | 3A8 | 53476 | 0.26 | (0.09) | 0.21 | (0.22) | 0.00 | (0.00) | 0.00 | (0.00) |

Table S1. Continued.

| | | | | | | |
|---|---|---|---|---|---|---|
| Df(1)ED6584 | 3A8 - 3B1 | 49222 | 0.40 ( 0.24 ) | 0.31 ( 0.11 ) | 0.00 ( 0.00 ) | 0.00 ( 0.00 ) |
| Df(1)ED6630 | 3B1 - 3C5 | 351370 | 0.44 ( 0.22 ) | 0.26 ( 0.09 ) | 0.05 ( 0.11 ) | 0.00 ( 0.00 ) |
| Df(1)ED6712 | 3D3 - 3F1 | 357080 | 0.13 ( 0.08 ) | 0.20 ( 0.21 ) | 0.00 ( 0.00 ) | 0.00 ( 0.00 ) |
| Df(1)ED6727 | 4B6 - 4D5 | 585887 | 0.46 ( 0.28 ) | 0.18 ( 0.14 ) | 0.00 ( 0.00 ) | 0.00 ( 0.00 ) |
| Df(1)ED6802 | 5A12 - 5D1 | 285900 | 0.63 ( 0.24 ) | 0.30 ( 0.17 ) | 0.00 ( 0.00 ) | 0.00 ( 0.00 ) |
| Df(1)ED6829 | 5C7 - 5F3 | 451119 | 0.50 ( 0.19 ) | 0.12 ( 0.05 ) | 0.00 ( 0.00 ) | 0.00 ( 0.00 ) |
| Df(1)ED6849 | 5F3 - 6D3 | 452200 | 0.26 ( 0.12 ) | 0.03 ( 0.05 ) | 0.00 ( 0.00 ) | 0.00 ( 0.00 ) |
| Df(1)ED6878 | 6C12 - 6D8 | 103655 | 0.42 ( 0.22 ) | 0.27 ( 0.07 ) | 0.00 ( 0.00 ) | 0.00 ( 0.00 ) |
| Df(1)ED6906 | 7A3 - 7B2 | 210722 | 0.38 ( 0.08 ) | 0.05 ( 0.03 ) | 0.00 ( 0.00 ) | 0.01 ( 0.02 ) |
| Df(1)ED6940 | 36A10 - 36B1 | 297221 | 0.44 ( 0.16 ) | 0.42 ( 0.13 ) | 0.00 ( 0.00 ) | 0.00 ( 0.00 ) |
| Df(1)ED6957 | 8B6 - 8C13 | 243242 | 0.75 ( 0.19 ) | 0.58 ( 0.13 ) | 0.00 ( 0.00 ) | 0.00 ( 0.00 ) |
| Df(1)ED6989 | 8F9 - 9B1 | 383820 | 0.51 ( 0.18 ) | 0.13 ( 0.16 ) | 0.00 ( 0.00 ) | 0.00 ( 0.00 ) |
| Df(1)ED6991 | 8F9 - 9B4 | 524871 | 0.18 ( 0.17 ) | 0.18 ( 0.06 ) | 0.00 ( 0.00 ) | 0.00 ( 0.00 ) |
| Df(1)ED7005 | 9B1 - 9D3 | 513509 | 0.15 ( 0.08 ) | 0.06 ( 0.05 ) | 0.00 ( 0.00 ) | 0.00 ( 0.00 ) |
| Df(1)ED7010 | 9D3 - 9D4 | 82437 | 0.11 ( 0.05 ) | 0.21 ( 0.14 ) | 0.00 ( 0.00 ) | 0.00 ( 0.00 ) |
| Df(1)ED7067 | 10B8 - 10C10 | 210959 | 0.38 ( 0.06 ) | 0.25 ( 0.17 ) | 0.00 ( 0.00 ) | 0.00 ( 0.00 ) |
| Df(1)ED7147 | 10D7 - 11A1 | 290417 | 0.20 ( 0.15 ) | 0.06 ( 0.05 ) | 0.00 ( 0.00 ) | 0.00 ( 0.00 ) |
| Df(1)ED7153 | 11A1 - 11B1 | 560373 | 0.42 ( 0.13 ) | 0.28 ( 0.09 ) | 0.00 ( 0.00 ) | 0.00 ( 0.00 ) |
| Df(1)ED7161 | 11A1 - 11B14 | 743779 | 0.34 ( 0.16 ) | 0.00 ( - ) | 0.00 ( 0.00 ) | 0.00 ( - ) |
| Df(1)ED7165 | 11B15 - 11E1 | 386346 | 0.44 ( 0.07 ) | 0.28 ( 0.15 ) | 0.00 ( 0.00 ) | 0.00 ( 0.00 ) |
| Df(1)ED7170 | 11B15 - 11E8 | 524724 | 0.57 ( 0.13 ) | 0.28 ( 0.12 ) | 0.00 ( 0.00 ) | 0.00 ( 0.00 ) |
| Df(1)ED7173 | 11B15 - 11F1 | 621133 | 0.16 ( 0.06 ) | 0.00 ( - ) | 0.00 ( 0.00 ) | 0.00 ( - ) |
| Df(1)ED7217 | 12A9 - 12C6 | 180238 | 0.30 ( 0.12 ) | 0.07 ( 0.05 ) | 0.00 ( 0.00 ) | 0.00 ( 0.00 ) |
| Df(1)ED7229 | 12E5 - 12F2 | 431710 | 0.80 ( 0.16 ) | 0.54 ( 0.16 ) | 0.03 ( 0.07 ) | 0.00 ( 0.00 ) |
| Df(1)ED7261 | 12F2 - 12F5 | 185603 | 0.31 ( 0.10 ) | 0.34 ( 0.16 ) | 0.00 ( 0.00 ) | 0.00 ( 0.00 ) |
| Df(1)ED7265 | 12F4 - 13A5 | 181838 | 0.49 ( 0.33 ) | 0.37 ( 0.22 ) | 0.00 ( 0.00 ) | 0.02 ( 0.05 ) |
| Df(1)ED7289 | 13A5 - 13A12 | 100973 | 0.53 ( 0.19 ) | 0.26 ( 0.20 ) | 0.00 ( 0.00 ) | 0.00 ( 0.00 ) |
| Df(1)ED7294 | 13B1 - 13C3 | 274883 | 0.41 ( 0.24 ) | 0.32 ( 0.09 ) | 0.00 ( 0.00 ) | 0.00 ( 0.00 ) |
| Df(1)ED7331 | 13C3 - 13F1 | 363268 | 0.46 ( 0.14 ) | 0.14 ( 0.02 ) | 0.00 ( 0.00 ) | 0.00 ( 0.00 ) |
| Df(1)ED7344 | 13E1 - 13F17 | 241694 | 0.34 ( 0.07 ) | 0.13 ( 0.06 ) | 0.01 ( 0.02 ) | 0.00 ( 0.00 ) |
| Df(1)ED7355 | 14A8 - 14B7 | 186930 | 0.21 ( 0.08 ) | 0.18 ( 0.11 ) | 0.10 ( 0.05 ) | 0.00 ( 0.00 ) |
| Df(1)ED7374 | 15A1 - 15E3 | 412445 | 0.62 ( 0.22 ) | 0.26 ( 0.09 ) | 0.00 ( 0.00 ) | 0.00 ( 0.00 ) |
| Df(1)ED7413 | 17D1 - 17F1 | 206484 | 0.39 ( 0.14 ) | 0.17 ( 0.10 ) | 0.00 ( 0.00 ) | 0.00 ( 0.00 ) |
| Df(1)ED7441 | 18A3 - 18C2 | 168474 | 0.20 ( 0.10 ) | 0.22 ( 0.09 ) | 0.00 ( 0.00 ) | 0.00 ( 0.00 ) |
| Df(1)ED7635 | 19A2 - 19C1 | 278714 | 0.41 ( 0.07 ) | 0.28 ( 0.16 ) | 0.04 ( 0.07 ) | 0.00 ( 0.00 ) |
| Df(1)ED7664 | 19F1 - 19F6 | 250376 | 0.43 ( 0.12 ) | 0.23 ( 0.05 ) | 0.00 ( 0.00 ) | 0.00 ( 0.00 ) |
| Df(1)ED11354 | 61B1 - 61C1 | 191859 | 0.37 ( 0.16 ) | 0.13 ( 0.09 ) | 0.00 ( 0.00 ) | 0.00 ( 0.00 ) |
| Df(1)ED11437 | 2F6 - 3A4 | 518880 | 0.32 ( 0.06 ) | 0.11 ( 0.10 ) | 0.00 ( 0.00 ) | 0.00 ( 0.00 ) |
| Df(1)ED12405 | 19C4 - 19E5 | 594760 | 0.41 ( 0.18 ) | 0.18 ( 0.08 ) | 0.00 ( 0.00 ) | 0.00 ( 0.00 ) |
| Df(1)ED12425 | 19E7 - 19F3 | 216238 | 0.41 ( 0.18 ) | 0.43 ( 0.21 ) | 0.00 ( 0.00 ) | 0.00 ( 0.00 ) |
| Df(1)ED12432 | 20C1 - 20C1 | 97858 | 0.39 ( 0.09 ) | 0.48 ( 0.19 ) | 0.34 ( 0.10 ) | 0.34 ( 0.09 ) |
| Df(1)ED13157 | 18F4 - 19C1 | 288549 | 0.44 ( 0.20 ) | 0.17 ( 0.06 ) | 0.05 ( 0.04 ) | 0.05 ( 0.11 ) |
| Df(1)ED13478 | 16F6 - 16F7 | 16605 | 0.42 ( 0.13 ) | 0.55 ( 0.12 ) | 0.00 ( 0.00 ) | 0.00 ( 0.00 ) |
| Df(1)ED14021 | 20C1 - 20E1 | 320915 | 0.46 ( 0.15 ) | 0.55 ( 0.16 ) | 0.00 ( 0.00 ) | 0.00 ( 0.00 ) |
